# Molecular Analysis of MgO Nanoparticle-Induced Immunity against Fusarium Wilt in Tomato

**DOI:** 10.3390/ijms24032941

**Published:** 2023-02-02

**Authors:** Yushi Takehara, Isamu Fijikawa, Akihiro Watanabe, Ayumi Yonemura, Tomoyuki Kosaka, Kosei Sakane, Kiyoshi Imada, Kazunori Sasaki, Hiroshi Kajihara, Shoji Sakai, Yoichi Mizukami, Muhammad Salman Haider, Sudisha Jogaiah, Shin-ichi Ito

**Affiliations:** 1 Graduate School of Sciences and Technology for Innovation, Yamaguchi University, Yamaguchi 753-8515, Yamaguchi, Japan; 2 Research Center for Thermotolerant Microbial Resources (RCTMR), Yamaguchi University, Yamaguchi 753-8515, Yamaguchi, Japan; 3 United Graduate School of Agricultural Sciences, Tottori University, Tottori 680-8553, Tottori, Japan; 4 Yamaguchi Prefectural Agriculture and Forestry General Engineering Center, 1-1-1 Ouchi-Hikami, Yamaguchi 753-0231, Yamaguchi, Japan; 5 Yamaguchi TLO, 2-16-1 Tokiwadai, Ube 755-8611, Yamaguchi, Japan; 6 Institute of Gene Research, Science Research Center, Yamaguchi University, 1-1-1 Minami-Kogushi, Ube 755-8505, Yamaguchi, Japan; 7 Institute of Horticultural Sciences, University of Agriculture, Faisalabad 38000, Pakistan; 8 Department of Environmental Science, Central University of Kerala, Tejaswini Hills, Kasaragod 671316, India

**Keywords:** nanoparticles, plant defense, glycine-rich protein, *Fusarium oxysporum*, *Solanum lycopersicum*, transcriptome, apoplast

## Abstract

Fusarium wilt, caused by *Fusarium oxysporum* f. sp. *lycopersici* (FOL), is a devastating soilborne disease in tomatoes. Magnesium oxide nanoparticles (MgO NPs) induce strong immunity against Fusarium wilt in tomatoes. However, the mechanisms underlying this immunity remain poorly understood. Comparative transcriptome analysis and microscopy of tomato roots were performed to determine the mechanism of MgO NP-induced immunity against FOL. Eight transcriptomes were prepared from tomato roots treated under eight different conditions. Differentially expressed genes were compared among the transcriptomes. The Kyoto Encyclopedia of Genes and Genomes enrichment analysis revealed that in tomato roots pretreated with MgO NPs, *Rcr3* encoding apoplastic protease and *RbohD* encoding NADPH oxidase were upregulated when challenge-inoculated with FOL. The gene encoding glycine-rich protein 4 (SlGRP4) was chosen for further analysis. *SlGRP4* was rapidly transcribed in roots pretreated with MgO NPs and inoculated with FOL. Immunomicroscopy analysis showed that SlGRP4 accumulated in the cell walls of epidermal and vascular vessel cells of roots pretreated with MgO NPs, but upon FOL inoculation, SlGRP4 further accumulated in the cell walls of cortical tissues within 48 h. The results provide new insights into the probable mechanisms of MgO NP-induced tomato immunity against Fusarium wilt.

## 1. Introduction

Global yield losses of important staple crops to pathogens and pests can reach 30%, with estimated costs to the global economy owing to lost food production in the hundreds of billions of dollars [1]. Therefore, control of plant diseases is an important and essential technology for staple crop production. Although various plant disease control technologies have been devised and developed, chemical fungicides are the most effective, labor-saving, and easy to use [2]. However, the large-scale use of chemical fungicides has caused problems that cannot be ignored, such as adverse effects on human health, emergence of fungicide-resistant microbes, burden on ecosystems, and food contamination owing to residual chemical fungicides [3]. Therefore, Integrated Pest Management (IPM), a pest management system that reduces the use of chemical fungicides by combining various pest control technologies to minimize risks to food and environmental safety, is being promoted internationally [4].

To accomplish IPM, it is important to develop pest control technologies other than chemical fungicides that have less impact on the environment. In this respect, the induction of disease resistance by plant immunity inducers, which suppress disease by activating the plant’s innate immune mechanism, is a promising disease control technology. In addition to their low environmental impact, plant immunity inducers have the advantage that there is almost no risk of the emergence of fungicide-resistant microbes, which is a problem with chemical fungicides [5]. Many plant immunity inducers, either chemically synthesized [5] or biologically derived [6], have been reported. Plant growth-promoting microorganisms (PGPR) found in the rhizosphere and root symbionts of plants are also known to induce plant immunity and have been applied in biocontrol [7].

Despite numerous reports, few plant immunity inducers have been put into practical use because of the large amount of time and expense required to develop plant immunity activators. In particular, there are very few plant immunity inducers developed and commercialized for vegetable diseases [8]. However, some known compounds and natural products, such as silicon [9] and chitosan [10], which are safe for humans and the environment, activate plant immunity. If these compounds can be applied for disease control, it would be advantageous for practical use because the development cost would be lower than that of new plant immunity activators. We found that magnesium oxide nanoparticles (MgO NPs), which are widely used as raw materials for fertilizers, soil conditioners, pharmaceuticals, and cosmetics, have an excellent plant immunity activation ability and have the potential to suppress bacterial wilt [11] and Fusarium wilt [12] in tomatoes.

Metal monoxides such as MgO NPs are known to produce reactive oxygen species (ROS) by interacting with biomolecules such as proteins, enzymes, and amino acids [13]. However, little is known about ROS production through interactions with plant-derived biomolecules. Imada et al. [11] showed that ROS are produced by the interaction of MgO NPs with plant polyphenols and speculated that the ROS generated in tomato roots treated with MgO NPs may be phenoxyl radicals produced by the deprotonation of phenolic hydroxyl radicals. In contrast, in *Arabidopsis* roots, ROS generation by MgO NPs has been suggested to involve NADPH oxidase (Rboh D), which acts in plant disease resistance responses [14].

MgO NPs are solid base catalysts of crystalline structured particles characterized by many edge/corner structures and reactive surface structures with a large surface area owing to their minute size [15]. MgO NPs with different surface structures can also be obtained depending on the calcination temperature [16]. We found that MgO NPs obtained by calcination at 700 °C induced significant ROS production in tomato and *Arabidopsis* roots [11,12,14]. It has been suggested that MgO NPs obtained by calcination at 700 °C have different surface structural characteristics from MgO NPs calcined at other temperatures (550, 900, 1000 °C), which may be involved in the interaction between MgO NPs and biomolecules [17].

Fusarium wilt of tomato is a soilborne disease caused by the filamentous fungus *Fusarium oxysporum* f. sp. *lycopersici* (FOL) that leads to significant economic losses to tomato production [18]. FOL survives for several years or more in the soil by forming thick-walled resting spores called chlamydospores, which germinate and invade the host through their roots and colonize xylems once the host is cultivated. The characteristic wilt symptoms appear as a result of severe water stress, mainly owing to vessel clogging caused by the growth of FOL. Wilting is most likely caused by a combination of pathogenic activities, such as the accumulation of fungal mycelia and/or toxin production [19]. Currently, control methods against Fusarium wilt mainly consist of soil fumigation with chemical fungicides and the use of resistant cultivars. Fumigation negatively affects the environment and human health. In addition, resistant varieties are losing their effectiveness owing to the continuous emergence of FOLs that overcome resistance. Therefore, it is highly desirable to establish alternative Fusarium wilt-control technologies. Biocontrol is a promising control method against Fusarium wilt, but it has the disadvantage that its effectiveness varies with the season, crop, and field [20].

In our previous report, we showed that, in tomato roots treated with MgO NPs, ROS are rapidly produced at the root tips where the FOL mycelium penetrates and at the vascular bundles through which FOL passes before entering the root xylem [12]. ROS strengthen the host cell wall [21] and serve as signals that mediate the activation of defense genes [22]. Therefore, ROS-mediated immunity against FOL may have been established in MgO NP-treated tomato roots before FOL infection. In fact, tomato seedlings irrigated at the base with a MgO NP suspension and grown for 7 days (pretreatment) did not develop Fusarium wilt until fruit harvest [12]. Moreover, because ROS play an important role in the adaptation process of plants to abiotic stresses [23], MgO NPs are suggested to be an abiotic stress for tomato roots. Furthermore, FOL infection is a biotic stressor in the tomato roots. Therefore, when tomato roots pretreated with MgO NPs were challenge-inoculated with FOL, they were subjected to both abiotic and biotic stresses. However, it is not known how tomato roots respond to these stressful conditions.

In this study, we aimed to determine the response of tomato roots to MgO NPs (short- and long-term treatments) as a nonbiotic stress and to FOL infection (with and without MgO NPs pretreatment) as a biotic stress. Transcriptome analysis was performed on tomato roots treated under eight different conditions. We selected the cell wall-localized glycine-rich protein (SlGRP4) gene from the genes whose expression was significantly upregulated in tomato roots pretreated with MgO NPs and challenge-inoculated with FOL as revealed by the transcriptome analysis, and immunostaining analysis confirmed the accumulation of SlGRP4 in the root tissue. In addition, we also performed microscopic observations to reveal the presence or absence of FOL proliferation in the roots of tomato plants pretreated with MgO NPs.

## 2. Results

### 2.1. RNA-seq Data Analysis

For transcriptome analysis, RNA was prepared from the roots of the following tomato plants and subjected to RNA sequencing: (i) those treated with distilled water for 1 h (H_2_O1h), (ii) those treated with MgO NPs for 1 h (MgO1h), (iii) those treated with distilled water for 7 days (H_2_O7d), (iv) those treated with MgO NPs for 7 days (MgO7d), (v) those treated with distilled water for 7 days and challenge-inoculated with FOL for 1 h (H_2_O7d + FOL1h), (vi) those treated with MgO NPs for 7 days and challenge-inoculated with FOL for 1 h (MgO7d + FOL1h), (vii) those treated with distilled water for 7 days and challenge-inoculated with FOL for 21 days (H_2_O7d + FOL21d), and (viii) those treated with MgO NPs for 7 days and challenge-inoculated with FOL for 21 days (MgO7d + FOL21d). After removing ambiguous nucleotides, 217,840,780 raw reads were obtained from the eight cDNA libraries. More than 96% of the high-quality reads in each sample could be mapped to the *Solanum lycopersicum* reference genome.

### 2.2. Identification of DEGs

**Figure 1:**
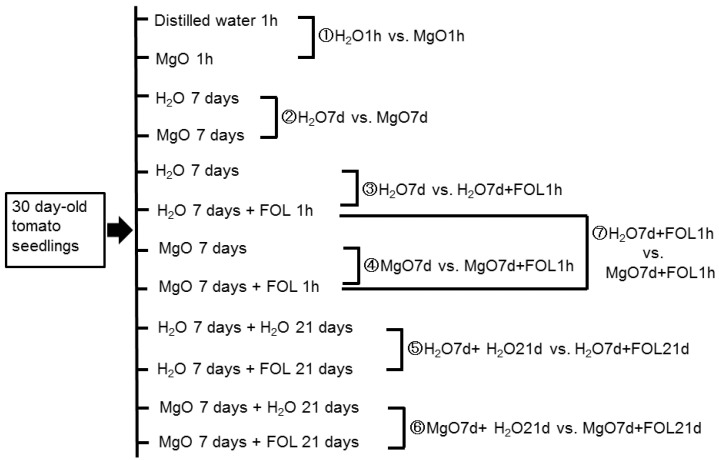
Seven comparative groups (①~⑦) of DEGs.

**Figure 2:**
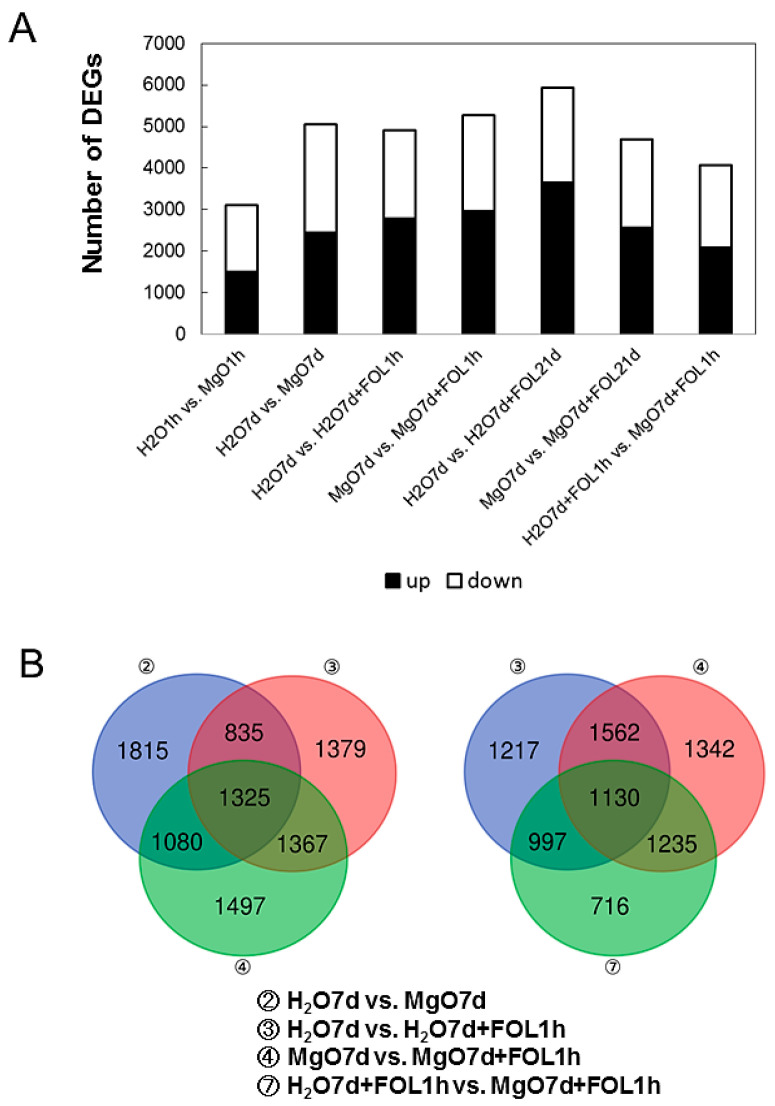
Number of DEGs in seven comparative groups. (**A**) Number of upregulated and downregulated genes. (**B**) Venn diagrams showing the number of DEGs in different three sample comparisons.

A total of 33,810 high-quality unigenes from tomatoes were mapped and annotated. DEGs were examined in the seven groups. (i) “H_2_O1h vs. MgO1h”, (ii) “H_2_O7d vs. MgO7d”, (iii) “H_2_O7d vs. H_2_O7d + FOL1h”, (iv) “MgO7d vs. MgO7d + FOL1h”, (v) “H_2_O7d + H_2_O 21 d vs. H_2_O7d + FOL21d”, (vi) “MgO7d + H_2_O21h vs. MgO7d + FOL21d”, and (vii) “H_2_O7d + FOL1h vs. MgO7d + FOL1h” (Figure 1). In tomato roots treated with MgO NPs for 1 h (H_2_O1h vs. MgO1h), 3098 DEGs with 1503 upregulated and 1595 downregulated genes were identified. In tomato roots treated with MgO NPs for 7 days (“H_2_O7d vs. MgO7d”), 5055 DEGs with 2446 upregulated and 2609 downregulated genes were identified. In tomato roots treated with H_2_O for 7 days and challenge-inoculated with FOL for 1 h (“H_2_O7d vs. H_2_O7d + FOL1h”), 4906 DEGs with 2786 upregulated and 2120 downregulated genes were identified. In tomato roots treated with MgO NPs for 7 days and challenge-inoculated with FOL for 1 h (“MgO7d vs. MgO7d + FOL1h”), 5269 DEGs with 2964 upregulated and 2305 downregulated genes were identified. In tomato roots treated with H_2_O for 7 days and challenge-inoculated with FOL for 21 days (“H_2_O7d vs. H_2_O7d + FOL21d”), 5923 DEGs with 3641 upregulated and 2282 downregulated genes were identified. In tomato roots treated with MgO NPs for 7 days and challenge-inoculated with FOL for 21 days (“MgO7d vs. MgO7d + FOL21d”), 4685 DEGs with 2557 upregulated and 2128 downregulated genes were identified. Expressed genes after FOL inoculation for 1 h with (“MgO7d + FOL1h”) and without (“H_2_O7d + FOL1h”) MgO NP pretreatment were also analyzed for DEGs. In this comparison group, 4078 DEGs with 2093 upregulated and 1985 downregulated genes were identified (Figure 2A). A comparison of genes whose expression changed significantly in response to FOL inoculation (1 h) with and without MgO NPs pretreatment revealed 716 genes specific to MgO NP-pretreated tomato roots (Figure 2B).

### 2.3. Gene Ontology (GO) and Kyoto Encyclopedia of Genes and Genomes (KEGG) Pathway Analyses of DEGs

To explore the biological functions of DEGs in the seven comparison groups (“H_2_O1h vs. MgO1h”, “H_2_O7d vs. MgO7d”, “H_2_O7d vs. H_2_O7d + FOL1h”, “MgO7d vs. MgO7d + FOL1h”, “H_2_O7d + H_2_O21h vs. H_2_O7d + FOL21d”, “MgO7d + H_2_O21h vs. MgO7d + FOL21d”, and “H_2_O7d + FOL1h vs. MgO7d + FOL1h”), the GO and KEGG enrichment analysis were conducted.

For GO analysis, the DEGs in the “H_2_O1h vs. MgO1h” group were classified into 151 functional terms, namely, 84 terms in biological process (BP), 9 in chemical component (CC), and 58 in molecular function (MF). The top 10 GO terms enriched for the upregulated genes in the BP category are listed in Table S1. The total DEGs in the “H_2_O7d vs. MgO7d” group were classified into 251 functional terms, namely, 116 terms in BP, 30 in CC, and 105 in MF. The top 10 GO terms enriched for the upregulated genes in the BP category are listed in Table S2. The DEGs of the “H_2_O7d vs. H_2_O7d + FOL1h” group were classified into 264 functional terms, namely, 162 terms in BP, 22 in CC, and 80 in MF. The top 10 GO terms enriched for the upregulated genes in the BP category are listed in Table S3. The DEGs of the “MgO7d vs. MgO7d + FOL1h” group were classified into 354 functional terms, namely, 218 terms in BP, 19 in CC, and 117 in MF. The top 10 GO terms enriched for the upregulated genes in the BP category are listed in Table S4. The DEGs of the “H_2_O7d+ H_2_O21h vs. H_2_O7d + FOL21d” group were classified into 334 functional terms, namely, 194 terms in BP, 44 in CC, and 96 in MF. The top 10 GO terms enriched for the upregulated genes in the BP category are listed in Table S5. The DEGs of the “MgO7d + H_2_O21h vs. MgO7d + FOL21d” were classified into 266 functional terms: 143 terms in BP, 31 in CC, and 92 in MF. The top 10 GO terms enriched for the upregulated genes in the BP category are listed in Table S6. The DEGs of the “H_2_O7d + FOL1h vs. MgO7d + FOL1h” were classified into 263 functional terms: 145 terms in BP, 21 in CC, and 97 in MF. The top 10 GO terms enriched for the upregulated genes in the BP category are listed in Table S7. The “H_2_O1h vs. MgO1h” comparison group showed enrichment of GO terms related to terpenoid and isoprenoid biosynthesis and metabolic process (Table S1), whereas the other six comparison groups were enriched in similar GO terms (Tables S2–S7).

KEGG enrichment analysis for the seven comparison groups was performed on 11922 of the 33811 tomato genes linked to Ko numbers (KEGG ortholog numbers) in the KEGG database. KEGG pathways enriched for the seven comparison groups are shown in the Supplementary Materials (Figures S1–S7). The top 10 pathways enriched in each comparative group are shown in Table 1, Table 2, Table 3, Table 4, Table 5, Table 6 and Table 7.

KEGG enrichment pathways of the seven comparative groups were analyzed. We particularly focused on three KEGG enrichment pathways, “MAPK signaling pathway-plant”, “Plant hormone signal transduction”, and “Plant-pathogen interaction”, because they were highly enriched in all comparative groups.

**Figure 3:**
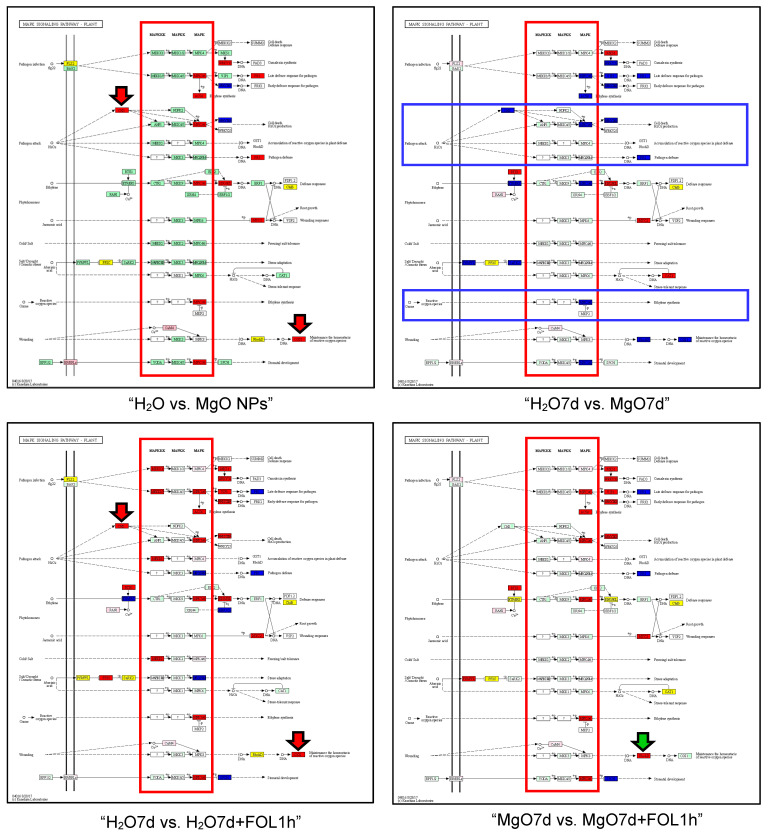

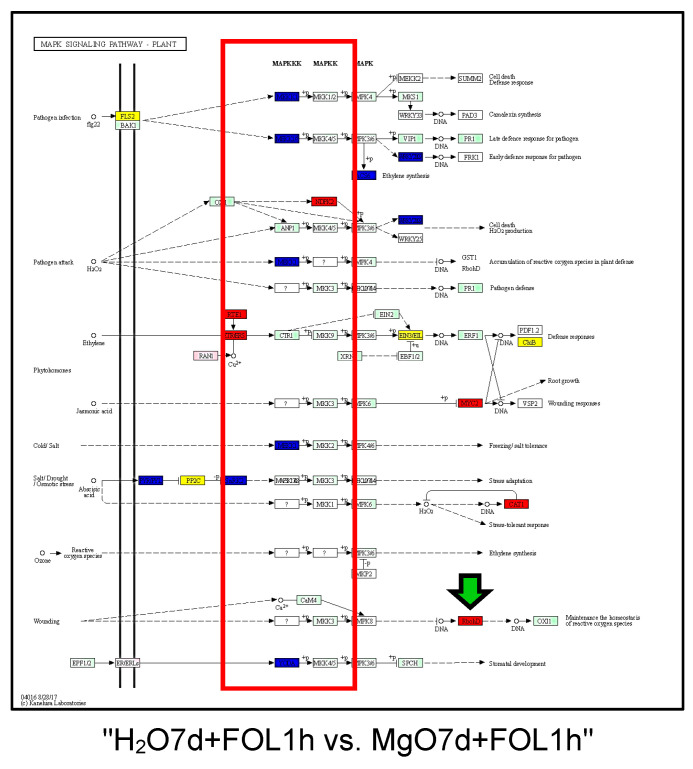
KEGG enrichment pathway: MAPK signaling pathway-plant. The red and blue boxes represent genes that are upregulated and downregulated, respectively. The yellow boxes represent genes that are both upregulated and downregulated. The red frames indicate MAPKKK, MAKK, and MAPK genes, from left to right. The blue frames indicate pathways with reduced expression. The red arrows indicate the upregulated *OXI1* gene. The green arrows show the upregulated *RbohD* gene. Black solid arrows indicate stimulation/activation. Black dashed arrows indicate indirect effect.

For “MAPK signaling pathway-plant”, genes involved in the following pathways were upregulated in each comparison group: “Pathogen infection”, “Pathogen attack”, “Ethylene”, “Jasmonic acid”, “Reactive oxygen species”, “Wounding” in the “H_2_O1h vs. MgO1h” group; “Ethylen” and “Jasmonic acid” in the “H_2_O7d vs. MgO7d” group; “Pathogen infection”, “Pathogen attack”, “Ethylene”, “Jasmonic acid”, “Cold/Salt”, “Abscisic acid”, “Reactive oxygen species“, and “Wounding” in the “H_2_O7d vs. MgO7d” group; “Pathogen infection”, “Pathogen attack”, “Ethylene”, “Jasmonic acid”, “Cold/Salt”, “Abscisic acid”, “Reactive oxygen species”, and “Wounding” in the “H_2_O7d vs. H_2_O7d + FOL1h” group; “Pathogen infection”, “Pathogen attack”, “Ethylene”, “Jasmonic acid”, “Cold/Salt”, “Abscisic acid”, “Reactive oxygen species”, and “Wounding” in the “MgO7d vs. MgO7d + FOL1h” group; “Pathogen infection”, “Pathogen attack”, “Ethylene”, “Jasmonic acid”, “Cold/Salt”, “Abscisic acid”, “Reactive oxygen species”, and “Wounding” in the “H_2_O7d vs. H_2_O7d + FOL21d” group; “Pathogen infection”, “Pathogen attack”, “Jasmonic acid”, and “Abscisic acid” in the “MgO7d vs. MgO7d + FOL21d” group; and “Ethylene” and “Abscisic acid” in the “H_2_O7d + FOL1h vs. MgO7d + FOL1h” group. Conversely, in the “H_2_O7d vs. MgO7d” group, genes involved in “Pathogen attack” and “Reactive oxygen species” pathways were downregulated (Figure 3, blue frame). In addition, MAPK genes were downregulated in this comparison group. In the “H_2_O1h vs. MgO1h” and “MgO7d vs. MgO7d + FOL1h” groups, only MAPK genes were upregulated among MAPKKK, MAPKK, and MAPK genes, whereas in the “H_2_O7d vs. H_2_O7d + FOL1h” group, MAPKKK as well as MAPK genes were upregulated (Figure 3, red frame). *OXI1* encoding a serine/threonine kinase induced by ROS was upregulated in the “H_2_O1h vs. MgO1h” and “H_2_O7d vs. H_2_O7d + FOL1h” groups (Figure 3, red arrow). *RbhoD* encoding NADPH oxidase was upregulated in tomato roots pretreated with MgO NPs and challenge-inoculated with FOL. (“MgO7d vs. MgO7d + FOL1h” and “H_2_O7d + FOL1h vs. MgO7d + FOL1h” groups) (Figure 3, green arrow).

For “Plant hormone signal transduction”, genes involved in the following pathways were upregulated in each comparison group: “Cytokinine”, “Gibberellin”, “Ethylene”, “Jasmonic acid” in the “H_2_O1h vs. MgO1h” group; “Jasmonic acid” and “Salicylic acid” in the “H_2_O7d vs. MgO7d” group; “Gibberellin”, “Abscisic acid”, and “Jasmonic acid” in the “H_2_O7d vs. H_2_O7d + FOL1h” group; “Gibberellin” and “Jasmonic acid” in the “MgO7d vs. MgO7d + FOL1h”; “Auxin” and “Brassinosteroid” in the “H_2_O7d vs. H_2_O7d + FOL21d” group; “Auxin” and “Jasmonic acid” in the “MgO7d vs. MgOd + FOL21d” group; and “Jasmonic acid” in the “H_2_O7d + FOL1h vs. MgO7d + FOL1h” group. In the “H_2_O7d vs. MgO7d” group, “Cytokinine” and “Abscisic acid” pathways were downregulated (Figure S8).

For “Plant-pathogen interaction”, the following genes were significantly upregulated in each comparison group: cyclic nucleotide gated channels (*CNGCs*), *MPK3/6*, *WRKY25/33*, *PR1*, *Rd19*, and *KCS1/10* in the “H_2_O1h vs. MgO1h” group; *Rcr3*, *SGT1*, and *KCS1/10* in the “H_2_O7d vs. MgO7d” group; *CaMCML*, *MEK1*, *MPK3/6*, *WRKY25/33*, *WRKY29*, *PBS1*, *SGT1*, and *KCS1/10* in the “H_2_O7d vs. H_2_O7d + FOL1h” group; *Rcr3*, *FLS2*, *Rboh*, *CaMCML*, *MPK4*, *WRKY25/33*, *MPK3/6*, *WRKY29*, *RIN4*, *SGT*, and *KCS1/10* in the “MgO7d vs. MgO7d + FOL1h” group; *CaMCML*, *MPK3/6*, *WRKY25/33*, *PR1*, *RIN4*, *RPM1*, *Rd19* in the “H_2_O7d vs. H_2_O7d + FOL21d” group; *Rcr3*, *WRKY25/33*, *WRKY29*, *RPM1*, *Rd19*, and *KCS1/10* in the “MgO7d vs. MgO7d + FOL21d” group; and *Rcr3*, *Rboh*, *CaMCML*, *RIN4*, and *HSP90* in the “H_2_O7d + FOL1h vs. MgO7d + FOL1h” group.

**Figure 4:**
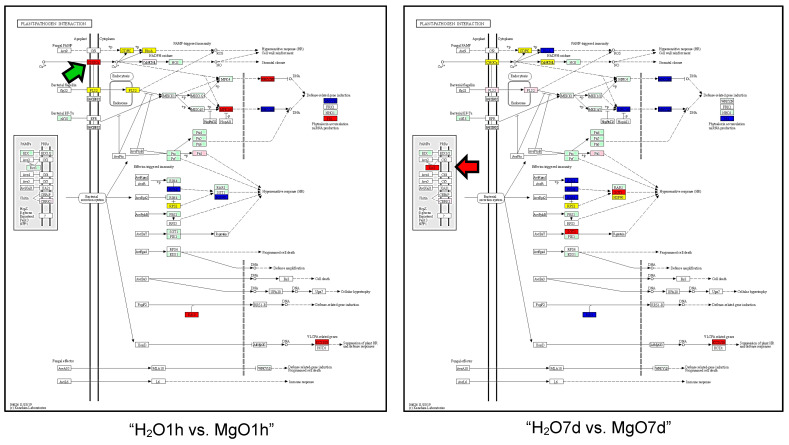

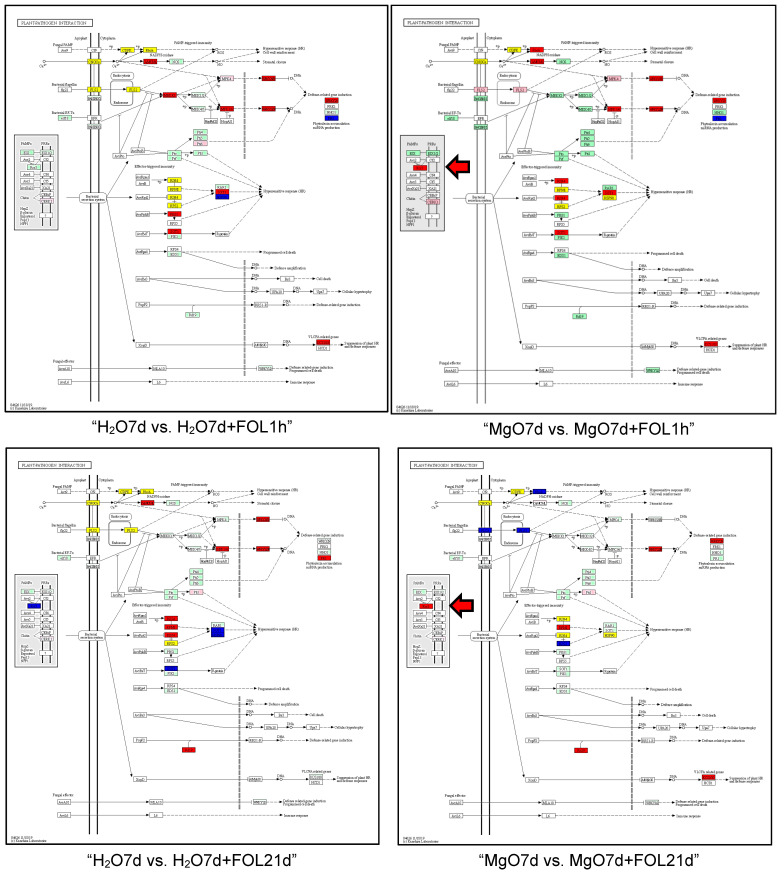

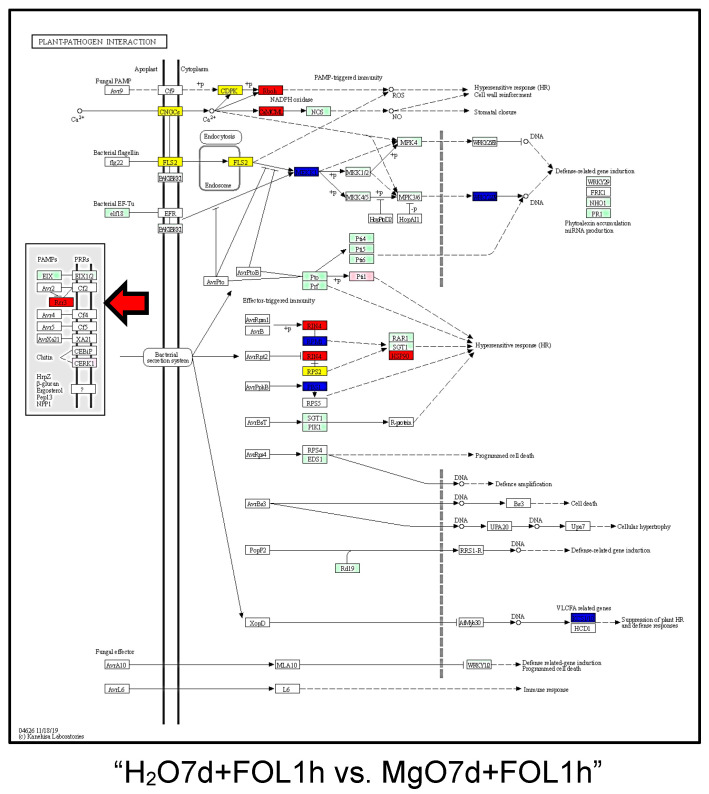
KEGG enrichment pathway: Plant-pathogen interaction. The red and blue boxes represent genes that are upregulated and downregulated, respectively. The yellow boxes represent genes that are both upregulated and downregulated. The red arrows show the upregulated *Rcr3* gene. The green arrow shows the upregulated *cyclic nucleotide gated channels* (*CNGCs*) gene. Black solid arrows indicate stimulation/activation. Black dashed arrows indicate indirect effect.

*Rcr3* gene encoding a papain-like apoplastic protease was commonly upregulated in the tomato roots treated with MgO NPs for 7 days (“H_2_O7d vs. MgO7d”, “MgO7d vs. MgO7d + FOL1h”, “MgO7d vs. MgO7d + FOL21d”, and “H_2_O7d + FOL1h vs. MgO7d + FOL1h” groups) (Figure 4, red arrow). The *cyclic nucleotide gated channels* (*CNGCs*) gene was upregulated only in tomato roots treated with MgO NPs for 1 h (“H_2_O1h vs. MgO1h” group) (Figure 4, green arrow).

### 2.4. Quantitative Real-Time PCR (qRT-PCR) Analysis

**Figure 5:**
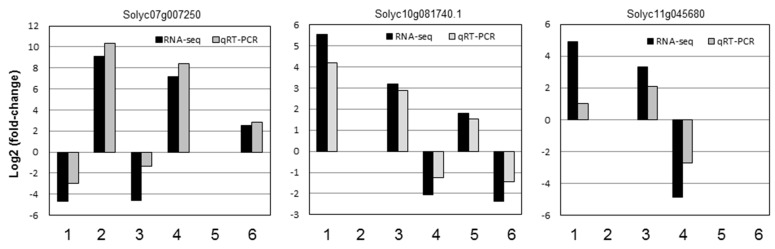
qRT-PCR analysis of DEGs in the seven comparative groups. 1, “H_2_O1h vs. MgO1h”; 2, “H_2_O7d vs. MgO7d”; 3, “H_2_O7d vs. H_2_O7d + FOL1h”; 4, “MgO7d vs. MgO7d + FOL1h”; 5, “H_2_O7d vs. H_2_O7d + FOL21d”; 6, “MgO7d vs. MgO7d + FOL21d”.

To verify the accuracy of RNA-seq in tomato plants, four DEGs were randomly selected for qRT-PCR analysis using specific primers. The results showed that the expression patterns of the four genes detected using qRT-PCR are consistent with the RNA-seq results (Figure 5).

### 2.5. Analysis of DEGs

The top 100 DEGs in each of the seven comparative groups are shown in the Supplementary Materials (Tables S8–S14).

**Figure 6:**
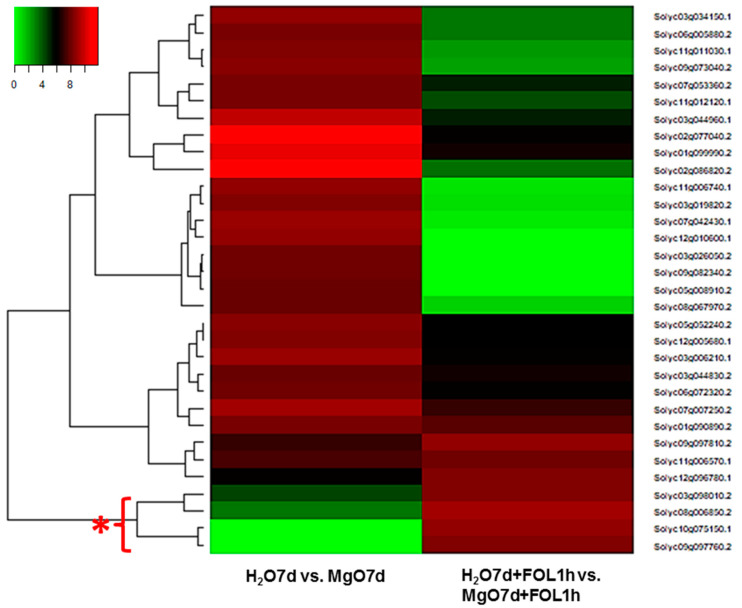
Heatmap analysis to detect genes that show remarkable upregulation (asterisk) when MgO-pretreated tomato plants were inoculated with *Fusarium oxysporum* f. sp. *lycopersici* compared to those before FOL inoculation.

To identify marker genes characterizing MgO NP-induced immunity against Fusarium wilt, we searched for genes that showed remarkable upregulation when MgO-pretreated tomato plants were inoculated with FOL compared to those before FOL inoculation. Four genes (*Solyc03g098010.2*, encoding acid phosphatase; *Solyc08g006850.2*, encoding a patatin-like phospholipase family protein; *Solyc10g075150.1*, encoding a nonspecific lipid-transfer protein; and *Solyc09g097760.2*, encoding a glycine-rich protein) showed such traits (Figure 6, asterisk). Among those genes, *Solyc09g097760.2* encoding glycine-rich protein (accession XP004248032.1; gene name: *SlGRP4* [24]), which has been suggested to be involved in Fusarium wilt resistance in tomato [25], was chosen for further analysis.

### 2.6. Expression Analysis of the SlGRP4 Gene

The transcript levels of *SlGRP4* were examined in the seven comparative groups. *SlGRP4* gene was differentially expressed with upregulation in the three comparative groups: “H_2_O1h vs. MgO1h” (response of roots to MgO NPs) (log_2_FC = 4.020), “MgO7d vs. MgO7d + FOL1h” (response of MgO NP-pretreated roots to FOL) (log_2_FC = 4.686), and “H_2_O7d + FOL1h vs. MgO7d + FOL1h” (differences in response between roots pretreated with and without MgO NPs to FOL) (log_2_FC = 8.495). No differences in the transcript levels were observed in “H_2_O7d vs. MgO7d” (response of roots to MgO NPs treatment for 7 days), “H_2_O7d vs. H_2_O7d + FOL1h” (response of roots to FOL inoculation), or “MgO7d vs. MgO7d + FOL21d” (response of MgO NPs-pretreated roots to long-term FOL inoculation) groups. In the H_2_O7d vs. H_2_O7d + FOL21d” group (susceptible response), the expression of *SlGRP4* was remarkably reduced (log_2_FC = −7.894).

**Figure 7:**
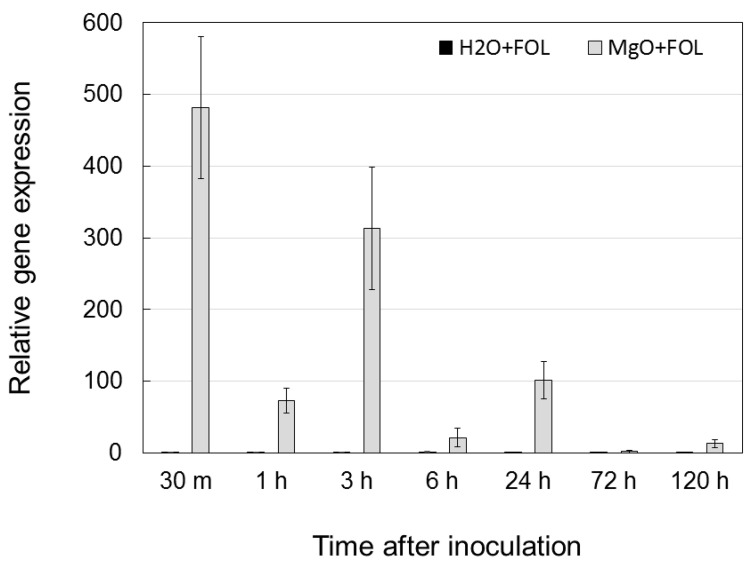
Time course of *SlGRP4* expression in tomato roots pretreated with MgO NPs and challenge-inoculated with *Fusarium oxysporum* f. sp. *lycopersici*.

To determine the expression pattern of *SlGRP4* in tomato roots pretreated with MgO NPs and challenge-inoculated with FOL, qRT-PCR was conducted with specific primers. In MgO NP-pretreated tomato roots, *SlGRP4* was highly expressed immediately (30 min) after FOL inoculation and decreased at 1 h. However, it then increased again at 3 h and 24 h and decreased at 72 h. In other words, multiple peaks of increased expression of *SlGRP4* were observed when MgO NP-pretreated roots were inoculated with FOL (Figure 7).

### 2.7. Prediction of Cis-Acting Elements in the SlGRP4 Gene

To understand the possible regulatory mechanisms of *SlGRP4*, the cis-acting elements encompassing 2000 bp 5′ upstream of the translation start site of the gene were analyzed using PlantCARE (https://bioinformatics.psb.ugent.be/webtools/plantcare/html/, accessed on 10 December 2022). Thirty-five cis-acting elements potentially involved in the regulation of *SlGRP4* were identified, including those that respond to phytohormones, such as abscisic acids, salicylic acid, and methyl jasmonate, and those recognized by MYC and MYB transcription factors (Figure S9).

### 2.8. Localization of SlGRP4 in Tomato Roots

**Figure 8:**
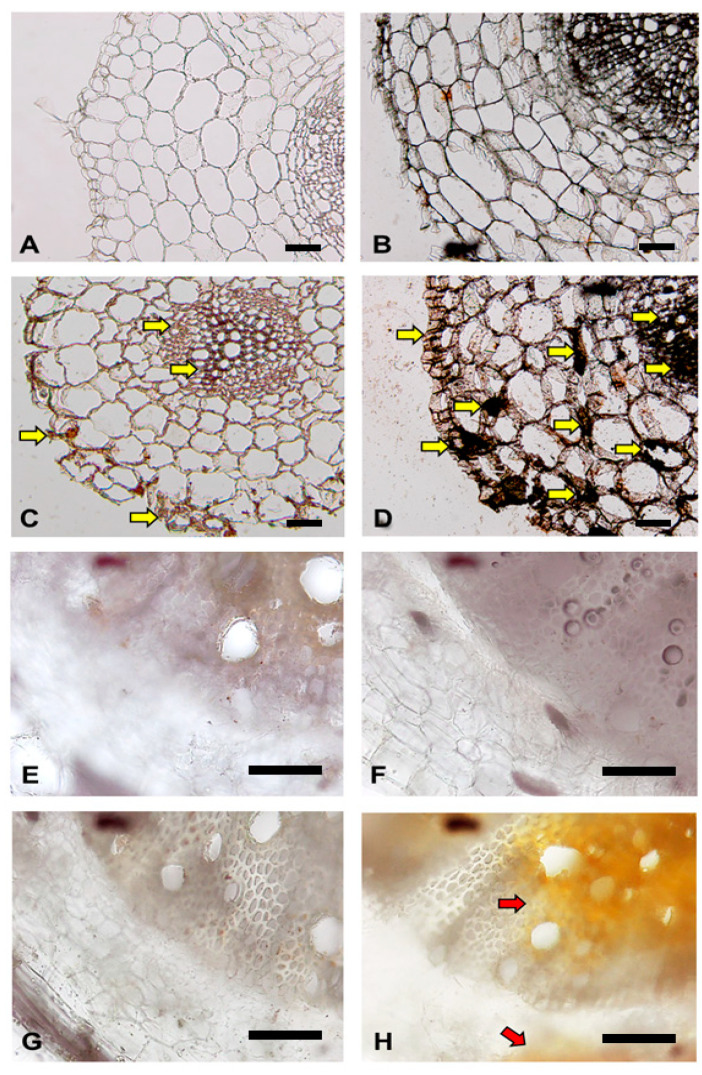
Immunomicroscopy of SlGRP4 protein (**A**–**D**) and detection of reactive oxygen species (ROS) (**E**–**H**) in tomato root tissues. (**A**) Control (no MgO treatment, no FOL inoculation), (**B**) tomato root 2 days after inoculation with FOL without MgO pretreatment, (**C**) tomato root treated with MgO NPs for 7 days (pretreatment only), (**D**) tomato root 2 days after inoculation with FOL with Mg NPs pretreatment, (**E**) control (no MgO treatment, no FOL inoculation), (**F**) tomato root 1 h after inoculation with FOL without MgO pretreatment, (**G**) tomato root treated with MgO NPs for 7 days (pretreatment only), and (**H**) tomato root 1 h after inoculation with FOL with Mg NPs pretreatment. Yellow arrows represent immuno-stained SlGRP4 proteins. Red arrows represent ROS (brown precipitate) detected by diaminobenzidine (DAB) staining. Scale bars represent 100 μm.

To localize the SlGRP4 protein expressed in tomato root tissues, immunostaining of tomato roots was performed using an anti-SlGRP4 antibody. SlGRP4 protein was detected in the cell walls of epidermal and vascular vessel cells of tomato roots treated with MgO NPs for 7 days (pretreatment only) (Figure 8C). In the root pretreated with MgO NPs and challenge-inoculated with FOL, SlGRP4 protein heavily accumulated in the cell walls of cortical tissue, epidermal tissue, and vascular vessel cells (Figure 8D). No accumulation of SlGRP4 protein was observed in tomato roots that were not treated with MgO NPs followed by FOL inoculation (Figure 8B). In the root pretreated with MgO NPs and challenge-inoculated with FOL, reactive oxygen species (ROS) were rapidly produced in cortical tissue and vascular vessel cells (Figure 8H).

### 2.9. Microscopic Observation of Tomato Root Tissue Pretreated with MgO NPs and Challenge-Inoculated with FOL

**Figure 9:**
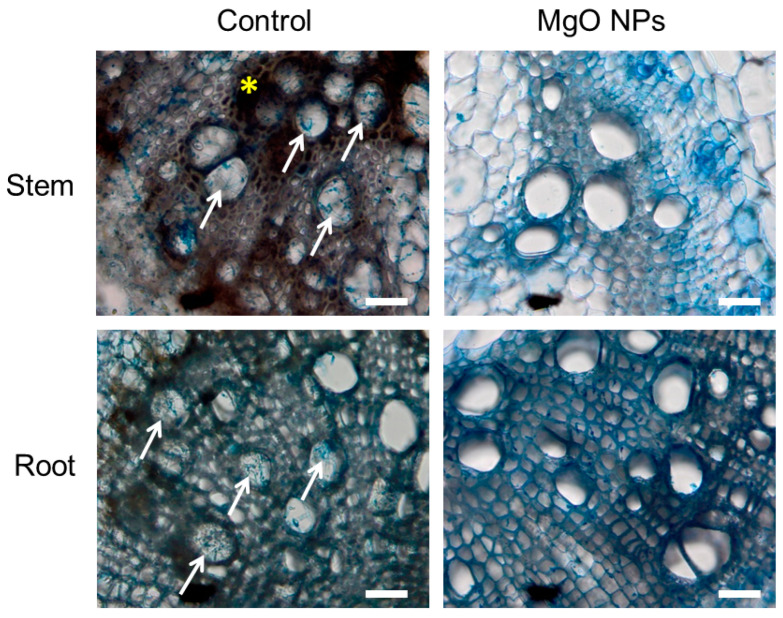
Microscopy images of tomato stems (10 cm above ground) and main roots 21 days after inoculation with *Fusarium oxysporum* f. sp. *lycopersici*. Cross sections of each tissue were stained with lactophenol cotton blue. White arrows point to the mycelial masses of *F. oxysporum* f. sp. *lycopersici*. Asterisk indicates the dark brown discoloration of vascular bundles. Scale bars represent 100 μm.

Tomato plants treated with and without MgO NPs were inoculated with FOL and grown for 21 days. Similar to the results reported in a previous study [12], tomato plants pretreated with MgO NPs were completely disease-free, even 21 days after FOL inoculation. In contrast, all tomato plants without MgO NPs pretreatment showed yellowing and wilting of leaves. Microscopy analysis revealed mycelial masses of FOL in the main roots and stems of the tomato plants that were not pretreated with MgO NPs (Figure 7, arrows). In addition, dark brown discoloration of vascular bundles, which is one of the symptoms of Fusarium wilt, was observed in the stems (Figure 9, asterisk). Few mycelia of FOL were observed in the roots and stems, and no discoloration of vascular bundles in the stem was observed in tomato plants pretreated with MgO NPs.

## 3. Discussion

In our previous report, we showed that when a MgO NPs suspension was drenched into the roots of tomato seedlings, ROS were produced in the tomato root tissue immediately after irrigation. In addition, tomato seedlings grown for 7 days after drenching (MgO NPs pretreatment) showed long-term immunity to FOL up to harvest, and the JA signaling pathway is essential for induction of this immunity [12]. However, gene expression in tomato roots immediately after MgO NPs treatment and in MgO NP-pretreated tomato roots is not known. We also did not know the gene expression patterns when MgO NP-pretreated tomato roots were inoculated with FOL, or the gene expression patterns in tomato roots with established immunity to FOL. Furthermore, how immunity to FOL is established in tomato roots is also unknown. This study aims to clarify these issues.

### 3.1. Response of Tomato Roots to MgO NPs

In this study, KEGG enrichment analysis for “MAPK signaling pathway-plant” of DEGs in the comparison group “H_2_O1h vs. MgO1h” showed that ROS-induced serine/threonine kinase *OXI1*, a serine/threonine kinase induced by ROS [26], was upregulated. *OXI1* is required for the full activation of *MPK3* and *MPK6*; *OXI1* is an essential part of the signal transduction pathway linking ROS signals to diverse downstream responses [26]. This suggests that *OXI1* induced by ROS is involved in the upregulation of *MPK3* and *MPK6*, which was shown in the roots 1 h after MgO NPs treatment, and further triggers downstream responses in which MPK3/6 acts. Interestingly, the gene (*Rboh*) for the enzyme (NADPH oxidase) that generates ROS [27] was not upregulated in “H_2_O1h vs. MgO1h”. However, the *CNGCs* gene, which triggers Ca^2+^ influx that activates Rboh [28], was upregulated, suggesting that Ca^2+^ influx may have activated Rboh and induced a ROS burst in the roots treated with MgO NPs. Among the DEGs of roots that were treated with MgO NPs for 1 h (“H_2_O1h vs. MgO1h”) (Table S8), *Solyc10g081740.1* encoding “calcium dependent protein kinase 1,” which is upregulated depending on Ca^2+^ [29], and *Solyc03g113940.2* encoding “calmodulin-binding protein 60-like G (CBP60g)” [30], which is involved in Ca^2+^ signaling in tomato plants [31], also suggest a Ca^2+^ influx in roots treated with MgO NPs. GO enrichment analysis of DEGs in “H_2_O1h vs. MgO1h” showed enrichment of GO terms involved in terpenoid production and metabolism. Because terpenoids mitigate the effects of oxidative stress by modulating the oxidative status of plants [32], a mitigation system for oxidative stress induced by MgO NPs would be activated in roots treated with MgO NPs.

In roots after 7 days of MgO NPs treatment (i.e., MgO NPs pretreatment: “H_2_O7d vs. MgO7d” group), genes involved in “Pathogen attack” and “Reactive oxygen species” pathways were downregulated in the “MAPK signaling pathway-plant” (Figure 3, blue frame). In addition, many genes involved in “Plant hormone signal transduction” and “Plant-pathogen interaction” pathways were downregulated. In contrast, *Rcr3*, which encodes a papain-like apoplastic protease, was upregulated. Rcr3 (required for *Cladosporium* resistance-3) is a secreted papain-like cysteine protease of tomato that acts as a coreceptor for the Cf-2 resistance protein to detect Avr2, a secreted apoplastic effector of the fungal pathogen *Cladosporium fulvum* [33]. Recognition of Avr2 by Cf-2 results in localized programmed cell death known as the hypersensitivity response (HR) [33]. *Rcr3* is upregulated by salicylic acid (SA) [34]. In fact, KEGG enrichment pathway “plant hormone signal transduction” analysis showed that the SA pathway was upregulated only in the “H_2_O7d vs. MgO7d” group among the seven comparison groups in this study, suggesting the production of SA in tomato roots pretreated with MgO NPs.

### 3.2. Response of Tomato Roots to Fusarium oxysporum

Many pathways and genes in the three KEGG enrichment pathways, “MAPK signaling pathway-plant”, “Plant hormone signal transduction”, and “Plant-pathogen interaction” were enriched in both the “H_2_O + FOL1h” and “MgO + FOL1h” groups. However, in “MAPK signaling pathway-plant” pathways, tomato roots pretreated with MgO NPs followed by FOL inoculation for 1 h (“MgO7d + FOL1h”) showed that only MAPK genes were upregulated among MAPKKK, MAPKK, and MAPK genes, whereas tomato roots pretreated with H_2_O followed by FOL inoculation (“H_2_O7d vs. H_2_O7d + FOL1h” group) showed that MAPKKK as well as MAPK genes were upregulated (Figure 3, red frame). As mentioned above, MAP3/6 genes were upregulated by the OXI1 transcription factor. *OXI1* was not upregulated in tomato roots pretreated with MgO NPs followed by FOL inoculation for 1 h, suggesting that OXI1-independent mechanisms for upregulation of MAPK3/6 genes were induced by MgO NPs pretreatment in the roots. In tomato roots pretreated with MgO NPs followed by FOL inoculation for 1 h (“MgO7d + FOL1h”), both *Rboh* encoding NADPH oxidase and *Rcr3* encoding a papain-like apoplastic protease were upregulated. In contrast, no upregulation of these genes was observed in tomato roots pretreated with H_2_O followed by FOL inoculation for 1 h (“H_2_O7d + FOL1h”). Corroborating the elevated expression of *Rboh*, ROS were rapidly produced in cortical and vascular tissues in roots pretreated with MgO NPs in response to FOL inoculation (Figure 8H); no ROS were detected when roots that had not been pretreated with MgO NPs were inoculated with FOL, suggesting that roots pretreated with MgO NPs were hypersensitive to FOL infection. The roles of NADPH oxidase and Rcr3 in suppressing FOL infection in tomato roots are unknown; however, both are involved in extracellular immunity [35]. Thus, their rapid expression within 1 h of inoculation seems to be important for extracellular immunity (apoplastic immunity) in MgO NP-induced immunity against FOL in tomatoes.

In tomato roots with no MgO NPs pretreatment 21 days after FOL inoculation, FOL had reached the stem xylems, and tomato plants showed symptoms of Fusarium wilt. In other words, the “H_2_O7d vs. H_2_O7d + FOL21d” group represents DEGs in the susceptible response. On the other hand, the “MgO7d vs. MgO7d + FOL21d” group represents DEGs in which MgO NPs-induced immunity has been established.

Comparing the two groups, the “Pathogen attack” pathway was enriched in the “MAPK signaling pathway-plant” in the “H_2_O7d vs. H_2_O7d + FOL21d” group (susceptible response). *MAP3/6* and *OXI1* were also upregulated (Figure 3). In addition, SA-inducible *PR1* was upregulated in the “Plant hormone signal transduction” pathways (Figure S8), suggesting that SA is produced in the roots with no MgO NPs pretreatment 21 days after FOL inoculation. This result is consistent with a report by Hernandez-Aparico et al. [36] that the activation of SA signaling occurs in susceptible responses. In contrast, in “MgO7d vs. MgO7d + FOL21d” (resistance response), *MAP3/6* and *OXI1* were not upregulated in the “MAPK signaling pathway-plant” pathways, but the apoplastic protease *Rcr3* was upregulated. This suggests the involvement of apoplastic immunity in the MgO NP-induced immunity against FOL.

### 3.3. SlGRP4

In the present study, we revealed that the *SlGRP* gene was rapidly upregulated within 1 h when tomato roots were treated with MgO NPs and when tomato roots pretreated with MgO NPs were inoculated with FOL, suggesting that *SlGRP* is upregulated in response to both abiotic (MgO NPs) and biotic (FOL) stresses. Cis-acting element analysis of the *SlGRP* gene revealed 35 cis-acting elements, including those that respond to phytohormones such as abscisic acids, SA, and methyl jasmonate, and those recognized by MYC and MYB transcription factors. We concluded that the *SlGRP* gene is involved in many stress responses. Increased GRP expression has been reported in response to abiotic stresses, such as salt, cold, heat, and injury, as well as biotic stress, including fungal infection [37].

GRPs are characterized by high glycine content and the presence of conserved segments including glycine-containing structural motifs of repetitive amino acid residues [38] and are classified into five classes (I to V) based on their general structure, taking into consideration the arrangement of the glycine repeats as well as the presence of conserved motifs [39]. The predicted amino acid sequence of SlGRP contains the Class V feature GGX/GXGX (where G represents glycine and X any amino acid). However, the function of class V GRPs is currently unknown.

GRPs localized in the cell walls of potato vascular bundles have been suggested to be involved in resistance to *Ralstonia solanacearum*, which causes vascular wilt disease such as FOL [24]. In the present study, it was shown that SlGRP4 accumulated in the cell walls of tomato vascular bundles and epidermal cells after MgO NPs treatment. Furthermore, inoculation of tomato roots pretreated with MgO NPs with FOL resulted in rapid and significant SlGRP4 upregulation, demonstrating that SlGRP4 protein accumulates in the cell walls of root cortex cells in addition to the cell walls of tomato vascular bundles and epidermal cells. After FOL invades tomato roots, the mycelium extends into the intercellular space of the cortex to reach the xylem vessels, during which many host defense reactions were observed [40]. Thus, SlGRP4 may play an important role in this process. In fact, in tomato roots pretreated with MgO NPs and challenge-inoculated by FOL, FOL mycelia were barely found in the tissues (Figure 9), suggesting that FOL infection of the root cortex and subsequent mycelial extension into xylems was strongly inhibited in tomato plants pretreated with MgO NPs even 21 days after FOL inoculation. However, because FOL is re-isolated from the roots of such plants [12], it is expected to be present and survive on the root surface. Further studies are warranted to examine the colonization of FOL on the surface of tomato roots pretreated with MgO NPs using GFP-labeled FOL.

### 3.4. Nanoparticles and Fusarium Wilt

The suppression of Fusarium wilt by nanoparticles, other than MgO NPs, has been demonstrated in metal oxide NPs such as CuO [41], ZnO [42], CaO [43], and Fe2O3 [44], and in metal NPs including silver [45] and manganese [46]. ROS induction after interaction with NPs has also been observed stably across plant species [47]. ROS induced by NPs acts as a signal for secondary metabolism, and secondary metabolites can act as antimicrobial compounds to protect plants from pathogenic microbes [47]. In the present study, GO enrichment analysis of DEGs in “H_2_O1h vs. MgO1h” showed that GO terms involved in the production and metabolism of terpenoids, which are materials for phytoalexins, were enriched (Table S1), suggesting that secondary metabolites accumulated in tomato roots pretreated with MgO NPs. This also suggests that tomato roots pretreated with MgO NPs may have already been ready to produce phytoalexins and other antimicrobial compounds before inoculation with FOL.

Present study revealed that rapid ROS production and SlGRP accumulation were induced in cortical tissue and vascular vessel cells of the roots pretreated with MgO NPs when challenge-inoculated with FOL (Figure 8), suggesting that ROS production may be associated with GRP accumulation. It is not known whether such rapid ROS production occurs in the roots pretreated with other NPs when challenge-inoculated with FOL. It is also unclear whether the “apoplastic immunity” suggested in the present study also occurs in other NPs-induced immunity against Fusarium wilt. Further studies are warranted to elucidate these points, which will provide important insights into Fusarium wilt resistance in tomato.

## 4. Materials and Methods

### 4.1. MgO NPs

The MgO NPs (Ube Material Industries, Yamaguchi, Japan) were produced by calcination of Mg (OH)_2_ at 700 °C. Water-dispersible MgO NP powder (KF-37) was prepared by Sankei Chemical (Tokyo, Japan).

### 4.2. Plant Material

Tomato plants (‘Momotaro’; Takii Seed, Kyoto, Japan) were grown in small pots (diameter, 9 cm; height, 7.6 cm) containing a vermiculite and perlite (1:1) mixture at 25 °C under a 12 h photoperiod with a photon flux density of 100 μmol·m^−2^·s^−1^ for 30 days. The 30-day-old tomato plants were subjected to MgO NP treatment, FOL inoculation experiments, and RNA sequencing, as described below.

### 4.3. Fungal Strains and Growth Conditions

The pathogen causing Fusarium wilt in tomatoes, FOL CK3–1 [48], was obtained from Dr. Takashi Tsuge (Nagoya University, Nagoya, Japan). A pure FOL culture was maintained on potato dextrose agar plates at 25 °C for 7 days. After incubation, fungal hyphal tips were obtained from the growing edge using a sterile sharp needle and transferred to 100 mL of potato dextrose broth (PDB). The broth was incubated at 25 °C with shaking at 120 rpm for seven days. The samples were filtered through three layers of gauze cloth and the filtrate was centrifuged at 3000× *g* for 5 min. The pellets were washed thrice and suspended in 20 mL of sterile distilled water. Conidial spore load was calculated using a hemocytometer and adjusted to 1 × 10^6^ conidia/mL for inoculation.

### 4.4. Inoculation Experiments

For treatment with MgO NPs, tomato plant pots were drenched by irrigation with 50 mL of 1% MgO NPs (KF-37) suspension or water (control), after which the plants were left to grow for seven days under the conditions described in Section 4.2. (MgO NPs pretreatment). Following the MgO NPs treatment, tomato roots were carefully washed with water to remove all surface MgO NPs. For FOL inoculation, roots were dipped in a fungal spore suspension or water (control) for 60 min. The inoculated plants were transferred to pots containing vermiculite and grown under preinoculation conditions. More than three tomato plants were used for each treatment, and all experiments were repeated at least thrice.

### 4.5. RNA Extraction

For RNA sequencing library construction, total RNA was extracted using Sepasol-RNA I Super G (Nacalai Tesque, Kyoto, Japan) from the roots of tomato plants with eight different treatments; (i) 30-day-old tomato plants were irrigated with water and left for 1 h (H_2_O1h), (ii) 30-day-old tomato plants were irrigated with 1% MgO NP suspension and left for 1 h (MgO1h), (iii) 30-day-old tomato plants were irrigated with water for 7 days (H_2_O7d), (iv) 30-day-old tomato plants were irrigated with 1% MgO NP suspension for 7 days (MgO NPs pretreatment only) (MgO7d), (v) tomato plants with no MgO NP pretreatment were inoculated with FOL for 1 h (H_2_O + FOL1h), (vi) (H_2_O7d + FOL21d), (vii) tomato plants with no MgO NP pretreatment were inoculated with FOL and grown for 21 days (H_2_O7d + FOL21d), and (viii) tomato plants pretreated with MgO NPs were inoculated with FOL and grown for 21 days (MgO7d + FOL21d). The total RNA concentration was determined using a Biospec-nano spectrophotometer (Shimadzu, Kyoto, Japan). More than three tomato plants were used for each treatment, and all experiments were repeated twice.

### 4.6. Preparation of cDNA Library and Sequencing

Aliquots (500 ng) of RNA were used for cDNA library preparation using an NEBNext Ultra II RNA Library Prep Kit for Illumina (NEB, Ipswich, MA, USA). cDNA libraries were sequenced using the Illumina MiniSeq platform to generate 75 bp single-end raw reads. Prior to assembly, raw data were filtered using a range of quality control measures. High-quality sequences were obtained from the original offline data sequences by removing the reads containing adapters and low-quality reads. All subsequent analyses were based on filtered clean data, which were aligned against the reference genome (GenBank accession number: GCA_000188115.3) using the CLC Genomics Workbench software ver. 12.0.3.

### 4.7. Screening of DEGs, Annotation, and GO and KEGG Analyses

The sequencing results were analyzed using CLC genomic workbench version 10.1.1. The reference genome sequence and annotation information of *Solanum lycopersicum* was obtained from NCBI (https://www.ncbi.nlm.nih.gov/genome/, accessed on 14 January 2020) and the used version was SL2.50. The value of unique exon read, which was obtained from the counted all mapped reads on exons, was used for gene expression profile analysis. Gene expression profiles were compared to find differentially expressed genes (DEGs) among different conditions using edgeR R package [49]. Genes with log_2_ (fold change) values greater than 1 or lower than −1 were assigned as DEGs. Gene ontology (GO) number assignment of each gene of *S. lycopersicum* SL2.50 was performed by blastp program [50] with the protein sequence data set of UniProt [uniprot_sprot, release-2019_10] (https://www.uniprot.org/, accessed on 25 October 2022). The cut-off criteria for assignment of a gene to homologous protein of UniProt database were >70% coverage and >25% identity, respectively. GO enrichment analysis of DEGs was performed using topGO R package [51] (https://bioc.ism.ac.jp/packages/3.12/bioc/html/topGO.html, accessed on 25 October 2022) with assigned GO number. GO terms with *p* values less than 0.01 were considered significantly enriched. KEGG orthology number assignment was performed on KofamKOALA website (https://www.genome.jp/tools/kofamkoala/, accessed on 25 October 2022). Pathway analysis was performed and visualized by KEGG (http://www.genome.jp/kegg/, accessed on 25 October 2022).

### 4.8. Gene Expression Analysis

Total RNA was extracted as described in Section 4.5. Reverse transcription was performed with 1 µg total RNA in a 20 µL reaction volume using ReverTra Ace qPCR RT Master Mix with gDNA Remover (Toyobo, Osaka, Japan) according to the manufacturer’s instructions. The primers used are listed in Table S15. cDNA was diluted (1:1), and 1 μL was used as a template in 20 μL of THUNDERBIRD SYBR qPCR Mix (Toyobo). The gene expression in each sample was normalized to that of tomato actin (GenBank accession No. FJ532351). Gene-specific primers were designed using Primer Express (Applied Biosystems, Foster City, CA, USA), based on sequences deposited in GenBank, to amplify 70–150 bp fragments (Table S10). Real-time quantitative polymerase chain reaction (RT-qPCR) analysis was performed using the Applied Biosystems’ QuantStudio Real-Time PCR system (Applied Biosystems). Relative quantification was performed using the ΔΔC_T_ method (Livak and Schmittgen, 2001 [52]).

### 4.9. Cis-Element Prediction

The generic file format of *Solanum lycopersicum* DC9.1 sequence (*SlGRP4*) [24] (accession: XP004248032.1) encompassing 2000 bp 5′ upstream of the translation start site was used to search the promoter sequence. Afterwards, the online program Plant CARE database (http://bioinformatics.psb.ugent.be/webtools/plantcare/html/, accessed on 10 December 2022) was used to identify the cis-acting elements of the sequence.

### 4.10. Microscopy

To detect FOL in root tissues, tomato roots pretreated with MgO NPs and challenge-inoculated with FOL were cut into 3 mm-long segments with a blade, which were prepared into sections for light microscopy using a freezing microtome (Nihon Khoki, Osaka, Japan). The sections were then stained with 1% cotton blue in lactophenol and observed under a BH2 light microscope (Olympus, Tokyo, Japan).

For immunostaining, tomato roots were cut into 3 mm-long segments with a blade and fixed in 5% glutaraldehyde in cacodylate buffer (pH 7.0). Fixed samples were immersed in *t*-butyl alcohol after dehydration through a graded series of *t*-butyl alcohol-ethanol and then embedded in paraffin. Transverse sections (14–18 m thick) were cut using a rotary microtome. Paraffin was removed using Histo-Clear (National Diagnostics, Atlanta, GA, USA), and sections were hydrated in a graded series of ethanol to water.

For immunohistochemical analysis for the localization of SlGRP4 in tomato root tissue, antibodies against a synthesized peptide fragment (CGGGYKPPHGEYKSPGGGYKPPH), designed based on the amino acid sequence of *Solanum lycopersicum* DC9.1 sequence (*SlGRP4*) [24] (accession: XP004248032.1), were raised in rabbits. The Vectastain ABC horseradish peroxidase staining kit (rabbit IgG) (Vector Laboratories, Burlingame, CA, USA) was used for immunohistochemical localization of SlGRP4 in tomato hypocotyl tissues with 3,3′-diaminobenzidine tetrahydrochloride (Wako Pure Chemical, Osaka, Japan) as the substrate following the manufacturer’s instructions. The sections were observed under an optical microscope (Olympus BH-2, Tokyo, Japan).

For ROS production in tomato root tissue, tomato roots were histochemically stained with 3′3′-diaminobenzidine (DAB) to visualize in situ ROS (hydrogen peroxide) accumulation. Tomato roots were immersed in DAB solution (2 mg/mL) for 15 min under vacuum, then root sections were cut with a razor blade and observed under a BH2 light microscope (Olympus).

## 5. Conclusions

Tomato seedlings grown for 7 days after drenching with MgO NPs suspension showed long-term immunity to Fusarium wilt of tomato up to harvest. The molecular mechanisms underlying this immune response are largely unknown. In this study, we attempted to identify the genes involved in MgO NP-induced immunity against Fusarium wilt using RNA-seq analysis. Our study revealed that *Rboh* encoding NADPH oxidase and *Rcr3* encoding a papain-like apoplastic protease were upregulated within 1 h after inoculation of FOL on roots pretreated with MgO NPs. This study also revealed that the *SlGRP4* gene was rapidly expressed in tomato roots treated with MgO NPs and its product SlGRR4 profusely accumulated in the cell walls of epidermal and vascular cells. In addition, upon FOL inoculation, SlGRR4 rapidly and greatly accumulated in the cell walls of cortical cells. Because Rboh, Rcr3, and cell wall-located GRPs are all involved in extracellular immunity (apoplastic immunity) in tomato plants, apoplastic immunity plays an important role in MgO NP-induced immunity against Fusarium wilt.

## Figures and Tables

**Table 1:** Top 10 pathways enriched in the “H_2_O1h vs. MgO1h” group.

Pathway	Up	Down	Total
Metabolic pathways	107	102	209
Biosynthesis of secondary metabolites	65	59	124
Carbon metabolism	15	11	26
Plant hormone signal transduction	12	9	21
Plant-pathogen interaction	10	10	20
MAPK signaling pathway-plant	12	7	19
Biosynthesis of amino acids	8	8	16
Biosynthesis of cofactors	11	4	15
Amino sugar and nucleotide sugar metabolism	8	3	11
Protein processing in endoplasmic reticulum	6	5	11

**Table 2:** Top 10 pathways enriched in the “H_2_O7d vs. MgO7d” group.

Pathway	Up	Down	Total
Metabolic pathways	160	213	373
Biosynthesis of secondary metabolites	90	139	229
Carbon metabolism	17	25	42
Plant hormone signal transduction	12	21	33
Biosynthesis of cofactors	13	20	33
Biosynthesis of amino acids	12	20	32
Plant-pathogen interaction	11	16	27
MAPK signaling pathway-plant	10	16	26
Amino sugar and nucleotide sugar metabolism	8	18	26
Starch and sucrose metabolism	13	10	23

**Table 3:** Top 10 pathways enriched in the “H_2_O7d vs. H_2_O7d + FOL1h” group.

Pathway	Up	Down	Total
Metabolic pathways	195	121	316
Biosynthesis of secondary metabolites	122	64	186
Plant hormone signal transduction	20	20	40
Carbon metabolism	21	16	37
MAPK signaling pathway-plant	21	11	32
Plant-pathogen interaction	19	10	29
Biosynthesis of amino acids	16	11	27
Biosynthesis of cofactors	16	9	25
Cysteine and methionine metabolism	12	9	21
Amino sugar and nucleotide sugar metabolism	14	6	20

**Table 4:** Top 10 pathways enriched in the “MgO7d vs. MgO7d + FOL1h” group.

Pathway	Up	Down	Total
Metabolic pathways	217	124	341
Biosynthesis of secondary metabolites	128	72	200
Carbon metabolism	33	18	51
Biosynthesis of amino acids	22	15	37
Plant hormone signal transduction	15	21	36
MAPK signaling pathway-plant	20	10	30
Plant-pathogen interaction	19	8	27
Biosynthesis of cofactors	17	9	26
Glycolysis/Gluconeogenesis	13	8	21
Amino sugar and nucleotide sugar metabolism	10	9	19

**Table 5:** Top 10 pathways enriched in the “H_2_O7d vs. H_2_O7d + FOL21d” group.

Pathway	Up	Down	Total
Metabolic pathways	256	182	438
Biosynthesis of secondary metabolites	160	105	265
Carbon metabolism	34	32	66
Biosynthesis of cofactors	32	19	51
Biosynthesis of amino acids	27	18	45
Ribosome	26	8	34
Glycolysis/Gluconeogenesis	18	14	32
Plant hormone signal transduction	16	15	31
Amino sugar and nucleotide sugar metabolism	22	8	30
Pyruvate metabolism	17	12	29

**Table 6:** Top 10 pathways enriched in the “MgO7d vs. MgO7d + FOL21d” group.

Pathway	Up	Down	Total
Metabolic pathways	163	159	322
Biosynthesis of secondary metabolites	88	108	196
Carbon metabolism	20	20	40
Plant hormone signal transduction	14	19	33
Biosynthesis of cofactors	14	15	29
Biosynthesis of amino acids	9	16	25
Starch and sucrose metabolism	11	13	24
MAPK signaling pathway-plant	13	10	23
Plant-pathogen interaction	13	10	23
Amino sugar and nucleotide sugar metabolism	8	14	22

**Table 7:** Top 10 pathways enriched in the “H_2_O7d + FOL1h vs. MgO7d + FOL1h” group.

Pathway	Up	Down	Total
Metabolic pathways	147	115	262
Biosynthesis of secondary metabolites	86	74	160
Carbon metabolism	27	12	39
Plant hormone signal transduction	14	18	32
Biosynthesis of amino acids	13	7	20
Biosynthesis of cofactors	11	7	18
Plant-pathogen interaction	10	11	21
MAPK signaling pathway-plant	11	11	22
Glycolysis/Gluconeogenesis	9	10	19
Starch and sucrose metabolism	7	9	16

## Data Availability

All raw reads were deposited in the DDBJ Sequence Read Archive (DRA) under accession number DRA015378.

## Supplementary Materials

The following supporting information can be downloaded at: https://www.mdpi.com/article/10.3390/ijms24032941/s1.
