# Supplementary material for: Molecular Analysis of MgO Nanoparticle-Induced Immunity against Fusarium Wilt in Tomato
Source: Int J Mol Sci. 2023 Feb 2;24(3):2941. doi: 10.3390/ijms24032941 (PMC9918173; doi:10.3390/ijms24032941)
Supplement: Supplementary file 1 [file ijms-24-02941-s001.zip › Table S15.pdf]

Table S15 Primers used in the present study.

Gene	Primer sequence
Solyc07g007250	F: 5'-GGTCAAAACCGGCGAGTTAG-3'
	R: 5'-CACTCTCCCGTCCTCAATCA-3'
Solyc10g081740.1	F: 5'-TGTTAGGGACCCGAAGCAAC-3'
	R: 5'-AGCGACTCAACACTGCAGAA-3'
Solyc11g045680	F: 5'-TAACCCCAACTACGACGTGC-3'
	R: 5'-ACTCCCAGCTGTTGTTGTTGT-3'
SlGRP4	F: 5'-ACGAACATACTGACGGACGT-3'
	R: 5'-TGCCACTACGTACGTATACCA-3'
