# Supplementary material for: Molecular Analysis of MgO Nanoparticle-Induced Immunity against Fusarium Wilt in Tomato
Source: Int J Mol Sci. 2023 Feb 2;24(3):2941. doi: 10.3390/ijms24032941 (PMC9918173; doi:10.3390/ijms24032941)
Supplement: Supplementary file 1 [file ijms-24-02941-s001.zip › Tables S1-S7.pdf]

Table S1. Top 10 GO terms enriched for upregulated genes in the biological process category in the "H_2O1h vs. MgO1h" group.

Term	Gene number	GO ID
Monoterpenoid metabolic process	25	GO:0016098
Monoterpenoid biosynthetic process	25	GO:0016099
Terpenoid metabolic process	136	GO:0006721
Isoprenoid metabolic process	152	GO:0006720
Terpenoid biosynthetic process	127	GO:0016114
Isoprenoid biosynthetic process	139	GO:0008299
(-)-Secologanin metabolic process	8	GO:1900992
(-)-Secologanin biosynthetic process	8	GO:1900994
Hormone biosynthetic process	123	GO:0042446
Beta-glucoside metabolic process	9	GO:1901804

Table S2. Top 10 GO terms enriched for upregulated genes in the biological process category in the "H_2O7d vs. MgO7d" group.

Term	Gene number	GO ID
Response to stress	1362	GO:0006950
Defense response	685	GO:0006952
Immune system process	192	GO:0002376
Immune response	189	GO:0006955
Response to biotic stimulus	532	GO:0009607
Innate immune response	161	GO:0045087
Response to nitrate	10	GO:0010167
Response to external stimulus	572	GO:0009605
Response to stimulus	2179	GO:0050896
Potassium ion homeostasis	7	GO:0055075

Table S3. Top 10 GO terms enriched for upregulated genes in the biological process category in the "H_2O7d vs. H_2O7d+FOL1h" group.

Term	Gene number	GO ID
Response to stimulus	2202	GO:0050896
Glutathione metabolic process	49	GO:0006749
Response to stress	1384	GO:0006950
Response to acid chemical	436	GO:0001101
Response to oxygen-containing compound	566	GO:1901700
Response to chemical	1034	GO:0042221
Fruit ripening	60	GO:0009835
Cellular modified amino acid metabolic process	67	GO:0006575
Ethylene metabolic process	37	GO:0009692
Ethylene biosynthetic process	37	GO:0009693

Table S4. Top 10 GO terms enriched for upregulated genes in the biological process category in the "MgO7d vs. MgO7d+FOL1h" group.

Term	Gene number	GO ID
Response to stimulus	2202	GO:0050896
Drug catabolic process	155	GO:0042737
Response to stress	1384	GO:0006950
Glutathione metabolic process	49	GO:0006749
Catabolic process	738	GO:0009056
Antibiotic catabolic process	80	GO:0017001
Response to oxidative stress	158	GO:0006979
Defense response	709	GO:0006952
Cellular modified amino acid metabolic process	67	GO:0006575
Response to acid chemical	436	GO:0001101

Table S5. Top 10 GO terms enriched for upregulated genes in the biological process category in "the H_2O7d+H_2O21d vs. $H_2O7d+FOL21d$" group.

Term	Gene number	GO ID
Hydrogen peroxide catabolic process	66	GO:0042744
Hydrogen peroxide metabolic process	73	GO:0042743
Carbohydrate metabolic process	518	GO:0005975
Cell wall organization or biogenesis	306	GO:0071554
Reactive oxygen species metabolism	87	GO:0072593
Cell wall organization	261	GO:0071555
External encapsulating structure organization and biogenesis	272	GO:0045229
Cell division	151	GO:0051301
Cellular polysaccharide metabolism	112	GO:0044264
Cell cycle	220	GO:0007049

Table S6. Top 10 GO terms enriched for upregulated genes in the biological process category in the "MgO7d vs. MgO7d+FOL21d" group.

Term	Gene number	GO ID
External encapsulating structure organization and biogenesis	272	GO:0045229
Cell wall organization or biogenesis	306	GO:0071554
Cell wall organization	261	GO:0071555
Hydrogen peroxide catabolic process	66	GO:0042744
Syncytium formation	7	GO:0006949
Hydrogen peroxide metabolic process	73	GO:0042743
Formation of an anatomical structure involved in morphogenesis	78	GO:0048646
Photomorphogenesis	18	GO:0009640
Phenylpropanoid catabolic process	14	GO:0046271
Lignin catabolic process	14	GO:0046274

Table S7. Top 10 GO terms enriched for upregulated genes in the biological process category in the "H_2O7d+FOL1h vs. MgO7d+FOL1h" group.

Term	Gene number	GO ID
Response to stress	1384	GO:0006950
Lipid catabolic process	98	GO:0016042
Catabolic process	738	GO:0009056
Response to stimulus	2202	GO:0050896
Drug catabolic process	155	GO:0042737
Organic acid transport	55	GO:0015849
Carboxylic acid transport	55	GO:0046942
Antibiotic catabolic process	80	GO:0017001
Defense response	709	GO:0006952
Cellular response to inorganic substance	18	GO:0071241
