# Supplementary material for: Molecular Analysis of MgO Nanoparticle-Induced Immunity against Fusarium Wilt in Tomato
Source: Int J Mol Sci. 2023 Feb 2;24(3):2941. doi: 10.3390/ijms24032941 (PMC9918173; doi:10.3390/ijms24032941)
Supplement: Supplementary file 1 [file ijms-24-02941-s001.zip › Tables S8-S14.pdf]

Table S8. Top 100 DEGs up-regulated in "H_2O1h vs. MgO1h" group.

Gene ID	log FC	Product
Solyc06g062540.2	9.039131826	Putative acid phosphatase
Solyc05g050350.1	7.689754473	Cyclic nucleotide gated channel
Solyc02g080010.2	7.395885806	Putative resistance protein with an antifungal domain
Solyc04g072070.2	7.329179469	WRKY transcription factor 16
Solyc09g011540.2	7.185733634	Glutathione S-transferase-like protein
Solyc11g068620.1	6.748695827	NAC-domain protein
Solyc11g012120.1	6.528537632	Plant-specific domain TIGR01615 family protein
Solyc03g093940.1	6.40442673	Myb-related transcription factor-like protein
Solyc08g068630.2	6.40442673	Decarboxylase family protein
Solyc01g005310.2	6.268625341	Dynamin like protein
Solyc02g067940.1	6.11869991	Unknown Protein
Solyc01g094240.2	5.951367786	Short-chain dehydrogenase/reductase
Solyc11g007310.1	5.951367786	Genomic DNA chromosome 3 P1 clone MCB17
Solyc11g073110.1	5.951367786	Auxin-regulated protein
Solyc02g093600.2	5.951367786	class I heat shock protein
Solyc03g096300.2	5.951367786	WUSCHEL-related homeobox 5
Solyc04g010010.1	5.951367786	Thioredoxin-like protein 1
Solyc07g056190.2	5.951367786	NBS-LRR class disease resistance protein
Solyc01g005500.2	5.762049575	3-phenylpropionate-dihydrodiol/cinnamic acid-dihydrodiol dehydrogenase
Solyc01g006080.1	5.762049575	Protein involved in mRNA turnover and stability (ISS)
Solyc01g105920.2	5.762049575	Beta myrcene/limonene synthase
Solyc12g077660.1	5.762049575	Set protein
Solyc12g056160.1	5.762049575	Cation/H(+) antiporter 18
Solyc04g015570.2	5.762049575	AN1-type zinc finger protein 2B
Solyc04g077300.2	5.762049575	Serine/threonine-protein kinase receptor
Solyc05g056330.2	5.762049575	Transposase (Fragment)
Solyc06g069870.2	5.762049575	Dehydration-responsive family protein
Solyc09g010720.2	5.762049575	WD-40 repeat family protein
Solyc10g081740.1	5.544081076	Calcium dependent protein kinase 1
Solyc12g017360.1	5.544081076	Glucan endo-1 3-beta-glucosidase 4
Solyc12g099130.1	5.544081076	MYB transcription factor
Solyc03g070390.2	5.544081076	Alpha-1 4-glucan-protein synthase
Solyc03g113940.2	5.544081076	Calmodulin-binding protein

Solyc06g083370.2	5.544081076	Os01g0917200 protein
Solyc07g040660.2	5.544081076	Unknown Protein
Solyc08g066350.1	5.544081076	Histidine phosphotransfer protein
Solyc08g083360.2	5.544081076	Ferredoxin
Solyc09g008080.2	5.544081076	Unknown Protein
Solyc09g083360.2	5.544081076	Basic helix-loop-helix protein
Solyc01g104320.2	5.287217459	PAP fibrillin
Solyc10g006110.2	5.287217459	EH-domain-containing protein 1
Solyc10g049750.1	5.287217459	DNA repair protein RAD16
Solyc12g011020.1	5.287217459	Had superfamily (Subfamily iiia) phosphatase
Solyc02g076940.1	5.287217459	Unknown Protein
Solyc02g084250.2	5.287217459	Unknown Protein
Solyc02g084680.2	5.287217459	Short internodes 2
Solyc02g093870.2	5.287217459	Histidine amino acid transporter
Solyc02g094310.1	5.287217459	Pentatricopeptide repeat-containing protein
Solyc03g007220.2	5.287217459	Membrane receptor-like protein 1
Solyc03g117260.1	5.287217459	Pentatricopeptide (PPR) repeat-containing protein-like
Solyc03g119370.1	5.287217459	MYB transcription factor
Solyc04g015030.2	5.287217459	Metal ion binding protein
Solyc05g008980.2	5.287217459	Receptor-like protein kinase
Solyc05g012350.2	5.287217459	Hydrolase alpha/beta fold family protein
Solyc06g009040.2	5.287217459	Glutathione S-transferase
Solyc06g062560.1	5.287217459	Putative phosphatase
Solyc06g068900.2	5.287217459	Nudix hydrolase 4
Solyc06g069170.2	5.287217459	Unknown Protein
Solyc06g083490.2	5.287217459	Tropinone reductase-like protein 16
Solyc07g017950.2	5.287217459	Ribulose bisphosphate carboxylase small chain
Solyc07g039570.2	5.287217459	Tropinone reductase-like protein 16
Solyc07g062020.1	5.287217459	Glucan endo-1 3-beta-glucosidase 4
Solyc09g005180.2	5.287217459	FAD-binding domain-containing protein
Solyc09g074530.2	5.287217459	Bidirectional sugar transporter SWEET
Solyc03g098010.2	5.231779762	Purple acid phosphatase
Solyc11g069750.1	5.128684192	Nitrate transporter
Solyc01g006520.2	4.97449634	Serine/threonine-protein kinase
Solyc01g006540.2	4.97449634	Lipoxygenase
Solyc01g007200.2	4.97449634	Unknown Protein

Solyc01g081620.2	4.97449634	Vacuolar sorting protein
Solyc01g109180.2	4.97449634	Long chain acyl-CoA synthetase 2
Solyc10g005330.2	4.97449634	Homeobox-leucine zipper protein ATHB-14
Solyc10g008170.2	4.97449634	26S proteasome regulatory subunit
Solyc10g008700.1	4.97449634	MYB transcription factor
Solyc10g044750.1	4.97449634	VHS and GAT domain protein
Solyc10g084810.1	4.97449634	UPF0161 protein yidD
Solyc11g045680.1	4.97449634	Ethylene-responsive transcription factor 4
Solyc11g070150.1	4.97449634	Histidine phosphotransfer protein
Solyc12g096850.1	4.97449634	WAT1-related protein
Solyc02g071060.2	4.97449634	Purine permease family protein
Solyc02g077190.1	4.97449634	Unknown Protein
Solyc02g084130.2	4.97449634	Unknown Protein
Solyc03g006050.2	4.97449634	Leucine rich repeat family protein
Solyc03g006960.2	4.97449634	Protein phosphatase 2C
Solyc03g082470.2	4.97449634	Receptor like kinase, RLK
Solyc03g116360.1	4.97449634	Regulator of chromosome condensation
Solyc03g118440.1	4.97449634	domain-containing protein
Solyc03g118960.1	4.97449634	Multidrug and toxin extrusion protein 1
Solyc04g050930.2	4.97449634	Violaxanthin de-epoxidase
Solyc04g058110.2	4.97449634	Serine/threonine-protein kinase
Solyc04g078520.2	4.97449634	Uncharacterized basic helix-loop-helix protein
Solyc04g079330.1	4.97449634	Carboxypeptidase
Solyc06g062440.2	4.97449634	Cc-nbs-lrr, resistance protein
Solyc06g062550.2	4.97449634	Phosphatase
Solyc06g065630.2	4.97449634	Flavin-containing monooxygenase
Solyc06g068750.2	4.97449634	Transferase transferring glycosyl groups
Solyc06g070960.1	4.97449634	ATP-binding cassette transporter sub-family A member 14
Solyc07g021030.1	4.97449634	Unknown Protein
Solyc07g047840.2	4.97449634	Unknown Protein
Solyc08g077070.2	4.97449634	Unknown Protein

Table S9. Top 100 DEGs up-regulated in "H_2O7d vs. MgO7d group.

Gene ID	log FC	Product
Solyc02g086820.2	11.943855	Carbonic anhydrase
Solyc02g077040.2	11.83853	Cathepsin B-like cysteine proteinase 5
Solyc01g099990.2	10.608447	F-box protein PP2-B1
Solyc03g044960.1	9.7496151	Coiled-coil domain-containing protein 109A
Solyc07g007250.2	9.1001538	Metallocarboxypeptidase inhibitor
Solyc07g042430.1	9.025332	Dimethylaniline monooxygenase
Solyc03g006210.1	8.9864137	Cathepsin B-like cysteine proteinase
Solyc12g010600.1	8.9052785	DNA-directed RNA polymerase subunit beta
Solyc03g034150.1	8.8412849	Homeobox leucine zipper protein
Solyc11g006740.1	8.7969891	F-box protein PP2-B1
Solyc09g073040.2	8.6553056	Unknown Protein
Solyc05g052240.2	8.6302774	Probable chalcone--flavonone isomerase 3
Solyc11g011030.1	8.5788795	Pto-responsive gene 1 protein
Solyc12g005680.1	8.5788795	Potassium transporter
Solyc03g019820.2	8.5788795	Tonoplast intrinsic protein 32
Solyc01g090890.2	8.4126915	Xenotropic and polytropic retrovirus receptor
Solyc11g012120.1	8.3527507	Protein of unknown function DUF506
Solyc06g005880.2	8.290211	Receptor like kinase, RLK
Solyc07g053360.2	8.290211	Seed biotin-containing protein SBP65
Solyc03g026050.2	8.2578943	Terminal flower 1
Solyc06g072320.2	8.1910045	8-amino-7-oxononanoate synthase
Solyc09g082340.2	8.1910045	Vicilin
Solyc03g044830.2	8.084469	Guanine nucleotide-binding protein G(I)/G(S)/G(T) subunit beta-2
Solyc05g008910.2	8.084469	Glutamine amidotransferase
Solyc08g067970.2	8.0471341	Unknown Protein
Solyc12g016220.1	7.9694346	Cc-nbs-lrr, resistance protein
Solyc12g042210.1	7.9694346	Ethylene responsive transcription factor 2a
Solyc03g020050.2	7.9694346	Proteinase inhibitor II
Solyc06g073080.2	7.9694346	Flavonol synthase/flavanone 3-hydroxylase
Solyc06g050410.1	7.707551	Ulp1 protease family C-terminal catalytic domain containing protein
Solyc03g111610.2	7.6588794	HAD-superfamily hydrolase subfamily IA variant 3 containing protein expressed

Solyc06g073030.1	7.6588794	Band 7 stomatin family protein
Solyc01g091870.2	7.6357621	Major facilitator superfamily domain-containing protein 8
Solyc08g083130.2	7.6085082	Homeobox leucine zipper protein
Solyc12g044950.1	7.5021616	Omega-6 fatty acid desaturase
Solyc02g031790.1	7.5021616	Receptor like kinase, RLK
Solyc11g006570.1	7.3571305	Inositol oxygenase
Solyc10g054070.1	7.3263214	Stylish 2b
Solyc04g064710.2	7.3263214	Alcohol dehydrogenase 2
Solyc03g113150.2	7.2625996	Pectate lyase
Solyc06g006070.1	7.2625996	LRR receptor-like serine/threonine-protein kinase
Solyc09g009560.1	7.2625996	Protein TERMINAL FLOWER 1
Solyc12g089300.1	7.1959328	SELF PRUNING 9D
Solyc09g097810.2	7.1586236	SAR8.2 protein
Solyc01g110700.2	7.1260353	Unknown Protein
Solyc08g007430.1	7.0603798	Nitrate transporter
Solyc11g071550.1	7.0525782	Glutamyl-tRNA(Gln) amidotransferase subunit A
Solyc06g068810.2	7.0525782	Pentatricopeptide repeat-containing protein At5g50990
Solyc07g063770.2	6.9751794	Serine/threonine-protein kinase
Solyc10g084960.1	6.8933916	Glutathione S-transferase-like protein
Solyc02g078130.2	6.8933916	Basic helix-loop-helix family protein
Solyc05g005330.2	6.8933916	Cc-nbs-lrr, resistance protein with an R1 specific domain
Solyc05g008950.2	6.806687	Receptor-like kinase
Solyc07g052380.2	6.806687	Protein DETOXIFICATION
Solyc08g048290.2	6.806687	Myo-inositol transporter 1
Solyc01g107680.2	6.7144365	ATP-dependent Clp protease ATP-binding subunit ClpX
Solyc01g108390.2	6.7144365	Proteinase inhibitor
Solyc11g013390.1	6.7144365	GrpE protein homolog
Solyc12g094730.1	6.7144365	AT-rich interactive domain-containing protein 3A
Solyc02g079260.1	6.7144365	Pentatricopeptide repeat-containing protein
Solyc09g091550.2	6.6161441	S-adenosyl-L-methionine: salicylic acid carboxyl methyltransferase
Solyc12g019180.1	6.6158818	Polygalacturonase 7
Solyc03g122070.1	6.6158818	Uncharacterized ABC transporter ATP-binding protein/permease
Solyc06g050970.1	6.6158818	Ulp1 protease family C-terminal catalytic domain containing protein

Solyc09g065580.2	6.6158818	Unknown Protein
Solyc05g007950.2	6.5100976	Ribonuclease T2
Solyc07g054610.2	6.5100976	F-box family protein
Solyc01g006650.1	6.3959388	Ethylene insensitive 3 class transcription factor
Solyc02g062130.2	6.3959388	Ferredoxin--NADP reductase
Solyc03g044600.1	6.3959388	Unknown Protein
Solyc04g010030.1	6.3959388	Thioredoxin-like protein 1
Solyc05g048830.2	6.3959388	MYB transcription factor
Solyc06g053440.2	6.3959388	Unknown Protein
Solyc06g071400.2	6.3959388	Bidirectional sugar transporter SWEET
Solyc06g071650.1	6.3959388	AP-1 complex subunit mu
Solyc09g092400.1	6.3959388	Ectonucleoside triphosphate diphosphohydrolase 5
Solyc01g006520.2	6.2719645	Serine/threonine-protein kinase
Solyc11g017280.1	6.2719645	Receptor like kinase
Solyc02g082630.2	6.2719645	Phosphoinositide phosphatase family protein
Solyc03g006980.2	6.2719645	Alpha-L-fucosidase 1
Solyc03g053110.2	6.2719645	Rhodanese-like family protein-like protein
Solyc03g062740.2	6.2719645	Unknown Protein
Solyc03g093340.2	6.2719645	Genomic DNA chromosome 5 TAC clone K11J9
Solyc03g119120.2	6.2719645	Pentatricopeptide repeat-containing protein
Solyc04g074840.2	6.2719645	Protein DETOXIFICATION
Solyc06g071610.1	6.2719645	Retinol dehydrogenase 12
Solyc07g040660.2	6.2719645	Unknown Protein
Solyc09g005160.1	6.2719645	Ubiquitin-protein ligase E3
Solyc09g055550.2	6.2719645	Unknown Protein
Solyc08g061020.1	6.152081	Nitrate transporter
Solyc12g096780.1	6.1378031	Mitochondrial trans-2-enoyl-CoA reductase
Solyc01g008830.1	6.1363267	L-ascorbate oxidase
Solyc02g087540.1	6.1363267	Cell division AAA ATPase family protein
Solyc03g070390.2	6.1363267	Alpha-1 4-glucan-protein synthase
Solyc04g005700.2	6.1363267	Major latex-like protein
Solyc05g006220.2	6.1363267	IAA-amino acid hydrolase
Solyc05g010230.1	6.1363267	Ulp1 protease family C-terminal catalytic domain containing protein
Solyc05g015060.2	6.1363267	26S protease regulatory subunit 6B homolog
Solyc08g081860.1	6.1363267	Unknown Protein

Solyc06g060310.2	6.0495863	Chlorophyllide a oxygenase

Table S10. Top 100 DEGs up-regulated in "H_2O7d vs. H_2O7d+FOL1h" group.

Gene ID	log FC	Product
Solyc01g097380.1	11.6831298	Aldo/keto reductase family protein
Solyc09g083360.2	11.00172063	Basic helix-loop-helix protein
Solyc12g099130.1	10.77752169	MYB transcription factor
Solyc08g068630.2	10.71557089	Decarboxylase family protein
Solyc11g068620.1	9.955549508	NAC-domain protein
Solyc07g056190.2	9.666365736	NBS-LRR class disease resistance protein
Solyc02g091820.1	9.622027098	Transcription factor
Solyc12g057080.1	9.496662649	Glycosyltransferase
Solyc10g005440.1	9.341239275	Serine/threonine-protein kinase
Solyc05g050350.1	9.341239275	Cyclic nucleotide gated channel
Solyc12g099120.1	9.266395839	MYB transcription factor
Solyc02g064830.2	9.103946033	Indole-3-acetic acid-amido synthetase GH3.8
Solyc01g108360.2	8.766469705	Copper ion binding protein
Solyc03g119370.1	8.465584234	MYB transcription factor
Solyc03g025640.1	8.287783883	Cytochrome P450
Solyc01g105920.2	8.249434497	Beta myrcene/limonene synthase
Solyc11g069890.1	8.210037814	BEL1-like homeodomain protein 8
Solyc04g007630.1	8.127862214	Metal ion binding protein
Solyc10g055760.1	8.040721684	NAC domain protein
Solyc03g117260.1	7.947977479	Pentatricopeptide (PPR) repeat-containing protein-like
Solyc04g051360.2	7.940898626	Ethylene responsive transcription factor 2b
Solyc02g080010.2	7.899269328	LK, Receptor like protein, putative resistance protein with an antifungal domain
Solyc04g072070.2	7.7966234	WRKY transcription factor 16
Solyc04g077300.2	7.74242516	Serine/threonine-protein kinase receptor
Solyc03g093940.1	7.68611109	Myb-related transcription factor-like protein
Solyc05g054380.1	7.566425881	Major allergen Mal d 1
Solyc09g091670.2	7.435906042	ATP-binding cassette transporter
Solyc02g086210.2	7.365933249	Receptor-like protein kinase
Solyc01g081620.2	7.292392908	Vacuolar sorting protein
Solyc12g006560.1	7.214901602	Early nodulin 93 protein

Solyc08g068610.2	7.175473762	Decarboxylase family protein
Solyc10g055740.1	7.143434293	Lysine/histidine transporter
Solyc08g036640.2	7.026189078	Protein TIFY 5A
Solyc01g087010.2	6.953808129	Acetyl xylan esterase A
Solyc04g077630.2	6.953808129	Serine carboxypeptidase 1
Solyc04g015030.2	6.855103485	Metal ion binding protein
Solyc04g015570.2	6.855103485	AN1-type zinc finger protein 2B
Solyc05g007950.2	6.855103485	Ribonuclease T2
Solyc06g069170.2	6.855103485	Unknown Protein
Solyc09g090900.2	6.855103485	3-isopropylmalate dehydratase large subunit, chloroplastic
Solyc03g082470.2	6.749146568	Receptor like kinase
Solyc06g075690.2	6.733197722	Auxin-regulated protein
Solyc01g100010.2	6.691838503	F-box protein PP2-B1
Solyc03g093130.2	6.66563602	Xyloglucan endotransglucosylase/hydrolase
Solyc03g115060.2	6.634786547	Unknown Protein
Solyc07g063720.1	6.634786547	Serine/threonine-protein kinase
Solyc04g025530.2	6.589069759	Glutamate decarboxylase
Solyc01g106830.2	6.584046166	Phytosulfokines 4
Solyc01g102610.2	6.510574873	Ferric reductase oxidase
Solyc12g014010.1	6.497564039	Glucosyltransferase
Solyc10g086280.1	6.414137264	Heavy metal-associated domain containing protein expressed
Solyc11g073110.1	6.374652809	Auxin-regulated protein
Solyc03g078470.2	6.374652809	Receptor-like kinase
Solyc03g097440.2	6.374652809	11-beta-hydroxysteroid dehydrogenase-like
Solyc03g113150.2	6.374652809	Pectate lyase
Solyc04g077340.2	6.374652809	Serine/threonine-protein kinase
Solyc02g091990.2	6.373456987	1-aminocyclopropane-1-carboxylate synthase 3
Solyc01g005500.2	6.22458026	3-phenylpropionate-dihydrodiol/cinnamic acid-dihydrodiol dehydrogenase
Solyc01g005940.2	6.22458026	Phytoene synthase 3
Solyc01g099060.1	6.22458026	GDSL esterase/lipase
Solyc01g104320.2	6.22458026	PAP fibrillin
Solyc01g110050.1	6.22458026	Purple acid phosphatase
Solyc03g031630.2	6.22458026	Calcium-binding EF-hand family protein-like
Solyc07g053230.2	6.22458026	Myb-related transcription factor

Solyc08g083360.2	6.22458026	Photosynthetic NDH subunit of subcomplex B 3, chloroplastic
Solyc04g074950.2	6.222875215	Ovarian cancer-associated gene 2 protein homolog
Solyc01g079200.2	6.12599649	Gibberellin 2-beta-dioxygenase 6
Solyc11g005860.1	6.107667645	Cysteine desulfurase
Solyc01g107500.2	6.057064811	Unknown Protein
Solyc12g097000.1	6.057064811	Tir, resistance protein fragment
Solyc05g025680.1	6.057064811	Respiratory burst oxidase protein B
Solyc06g083370.2	6.057064811	Peptidase
Solyc06g083930.1	6.057064811	LOB domain protein-like
Solyc07g008210.2	6.057064811	TPR domain protein
Solyc08g077070.2	6.057064811	Unknown Protein
Solyc09g073040.2	6.057064811	Unknown Protein
Solyc09g082670.2	6.057064811	Heat stress transcription factor A3
Solyc08g036620.2	5.948895523	Protein TIFY 5A
Solyc03g116100.2	5.920910801	MYB transcription factor
Solyc11g018800.1	5.918818816	Peroxidase
Solyc01g087590.2	5.914340389	Polyamine oxidase 1
Solyc10g078260.1	5.867511828	Peptidylprolyl isomerase
Solyc02g084250.2	5.867511828	Unknown Protein
Solyc03g096300.2	5.867511828	WUSCHEL-related homeobox 5
Solyc04g007390.2	5.867511828	Serine/threonine protein kinase family protein
Solyc05g010180.2	5.867511828	Carotenoid isomerase-like
Solyc06g064590.1	5.867511828	BHLH transcription factor
Solyc09g015070.2	5.867511828	Reductase 1
Solyc09g065560.2	5.867511828	Sulfate transporter
Solyc03g093080.2	5.866659984	Xyloglucan endotransglucosylase/hydrolase
Solyc07g056000.2	5.861470262	Xyloglucan endotransglucosylase/hydrolase
Solyc09g005560.2	5.851984488	Zinc finger and SCAN domain containing 29
Solyc02g092120.2	5.827135951	Putative phytosulfokine peptide
Solyc03g117860.2	5.827135951	IBR finger domain protein
Solyc08g082110.2	5.806336162	WRKY transcription factor-30
Solyc03g093120.2	5.790140297	Xyloglucan endotransglucosylase/hydrolase
Solyc08g059710.2	5.786538733	Genomic DNA chromosome 5 P1 clone MUK11
Solyc03g122340.2	5.785746036	Lipoxygenase
Solyc04g077270.2	5.785226347	Serine/threonine-protein kinase

Table S11. Top 100 DEGs up-regulated in "MgO7d vs. MgO7d+FOL1h" group.

Gene ID	log FC	Product
Solyc04g072280.2	9.706179594	Laccase
Solyc02g077040.2	9.360197895	Cathepsin B-like cysteine proteinase 5
Solyc03g020050.2	9.318442282	Proteinase inhibitor II
Solyc01g073890.2	9.275442034	CHP-rich zinc finger protein-like
Solyc03g020080.2	9.208438692	Proteinase inhibitor II
Solyc08g006850.2	9.185394412	Patatin
Solyc01g097380.1	9.064305465	Aldo/keto reductase family protein
Solyc10g075150.1	8.986452663	Non-specific lipid-transfer protein
Solyc12g088970.1	8.816883162	Cytochrome P450
Solyc10g005440.1	8.723987015	Serine/threonine-protein kinase
Solyc01g087280.1	8.658557356	Polygalacturonase QRT2
Solyc12g010020.1	8.362391427	Leucyl aminopeptidase
Solyc01g103630.2	8.091996099	Glyoxalase/bleomycin resistance protein/dioxygenase
Solyc12g057070.1	8.041558849	Glycosyltransferase
Solyc03g033790.2	7.989294211	Cell division protease ftsH homolog 3
Solyc12g099120.1	7.878717027	MYB transcription factor
Solyc12g096250.1	7.820078723	N-hydroxycinnamoyl/benzoyltransferase 5
Solyc03g025640.1	7.820078723	Cytochrome P450
Solyc02g080080.2	7.695128092	Receptor like protein, putative resistance protein with an antifungal domain
Solyc01g105890.2	7.628345364	Linalool synthase
Solyc07g005630.2	7.628345364	Unknown Protein
Solyc01g094250.2	7.407167672	Short-chain dehydrogenase/reductase
Solyc07g042430.1	7.407167672	Dimethylaniline monooxygenase
Solyc10g079860.1	7.325205398	Beta-1 3-glucanase
Solyc10g005390.2	7.238304721	Linalool/nerolidol synthase
Solyc06g083590.2	7.238304721	B3 domain-containing transcription factor ABI3
Solyc11g011030.1	7.22935831	Pto-responsive gene 1 protein
Solyc07g007250.2	7.145832172	Metallocarboxypeptidase inhibitor
Solyc09g098380.1	7.145832172	Amino acid transporter
Solyc12g049400.1	7.054884118	Protein TIFY 3B
Solyc01g100000.2	7.047023989	F-box protein PP2-B1
Solyc10g080670.1	7.047023989	Unknown Protein

Solyc06g072770.2	7.047023989	RNA recognition motif-containing protein
Solyc08g005530.2	6.940947741	Unknown Protein
Solyc06g076160.2	6.934771205	Cytochrome P450
Solyc10g079680.1	6.826448689	BHLH transcription factor-like
Solyc11g068970.1	6.826448689	Aluminum-activated malate transporter
Solyc02g064830.2	6.826448689	Indole-3-acetic acid-amido synthetase GH3.8
Solyc08g013670.2	6.826448689	Photosystem I reaction center subunit N, chloroplastic
Solyc08g016210.2	6.826448689	LRR receptor-like serine/threonine-protein kinase
Solyc01g101170.2	6.702072967	Viridiflorene synthase
Solyc10g078770.1	6.702072967	Protein LE25
Solyc12g096900.1	6.702072967	Tir, resistance protein fragment
Solyc05g055330.2	6.702072967	ATP-binding cassette transporter
Solyc06g072760.2	6.702072967	Syntaxin-21
Solyc09g005560.2	6.677563812	Zinc finger and SCAN domain containing 29
Solyc09g091670.2	6.574601186	ATP-binding cassette transporter
Solyc01g005900.2	6.565954416	Transferase family protein
Solyc02g089770.2	6.565954416	Dihydroflavonol-4-reductase
Solyc07g005680.2	6.565954416	Carboxypeptidase
Solyc05g014590.2	6.538579968	Transcription Factor
Solyc10g076660.1	6.460448949	Anthocyanidin synthase
Solyc01g101180.2	6.415642263	Viridiflorene synthase
Solyc05g053860.2	6.415642263	Solute carrier family 22 member 5
Solyc10g078220.1	6.247828153	Cytochrome P450
Solyc11g071550.1	6.247828153	Glutamyl-tRNA(Gln) amidotransferase subunit A
Solyc12g006560.1	6.247828153	Early nodulin 93 protein
Solyc03g006210.1	6.247828153	Cathepsin B-like cysteine proteinase
Solyc05g008940.2	6.247828153	Receptor-like kinase
Solyc06g072320.2	6.247828153	8-amino-7-oxononanoate synthase
Solyc07g007290.1	6.247828153	Dimethylaniline monooxygenase 5
Solyc07g008210.2	6.247828153	TPR domain protein
Solyc07g053230.2	6.247828153	Myb-related transcription factor
Solyc07g063600.2	6.247828153	Chlorophyll a-b binding protein, chloroplastic
Solyc04g025530.2	6.160209016	Glutamate decarboxylase
Solyc01g108360.2	6.141051913	Copper ion binding protein
Solyc10g055740.1	6.083395138	Lysine/histidine transporter
Solyc01g110050.1	6.057892549	Purple acid phosphatase

Solyc10g051120.1	6.057892549	Mitochondrial pyruvate carrier
Solyc12g005680.1	6.057892549	Potassium transporter
Solyc04g014900.2	6.057892549	Receptor like kinase
Solyc05g008960.2	6.057892549	Receptor-like protein kinase
Solyc06g005310.2	6.057892549	MYB transcription factor
Solyc07g008520.2	6.057892549	Peptide transporter
Solyc08g005120.2	6.057892549	Cinnamoyl-CoA reductase-like protein
Solyc09g083360.2	5.972512278	Basic helix-loop-helix protein
Solyc06g073080.2	5.963673731	Flavonol synthase/flavanone 3-hydroxylase
Solyc01g005040.2	5.839104862	CASP-like protein
Solyc10g079660.1	5.839104862	Importin alpha-1b subunit
Solyc11g005830.1	5.839104862	NifS-like protein Aminotransferase class V family
Solyc12g040800.1	5.839104862	C2 domain-containing protein
Solyc02g087450.2	5.839104862	Unknown Protein
Solyc03g098720.2	5.839104862	Kunitz trypsin inhibitor
Solyc03g111540.1	5.839104862	Receptor like protein, putative resistance protein with an antifungal domain
Solyc04g008140.1	5.839104862	Cc-nbs-lrr, resistance protein
Solyc04g071680.1	5.839104862	Receptor like kinase
Solyc05g008030.1	5.839104862	Unknown Protein
Solyc06g034000.1	5.839104862	Myb-related transcription factor-like protein
Solyc06g074100.1	5.839104862	Aluminum-activated malate transporter
Solyc07g008690.2	5.839104862	Alpha-humulene/(-)-(E)-beta-caryophyllene synthase
Solyc07g014750.1	5.839104862	Unknown Protein
Solyc07g054960.1	5.839104862	Myb-related transcription factor
Solyc08g014230.2	5.839104862	Isopropylmalate synthase
Solyc08g016270.1	5.839104862	LRR receptor-like serine/threonine-protein kinase
Solyc09g014860.2	5.839104862	Os03g0816700 protein
Solyc11g005860.1	5.83572275	Cysteine desulfurase
Solyc01g097240.2	5.777089628	Pathogenesis-related protein P2
Solyc02g092110.2	5.66990773	Putative phytosulfokine peptide
Solyc02g091820.1	5.5941532	Transcription factor

Table S12. Top 100 DEGs up-regulated in "H_2O7d+H_2O21d vs. H_2O7d+FOL21d" group.

Gene ID	log FC	Product
Solyc03g116060.2	13.7472618	Gibberellin-regulated protein 4
Solyc12g006680.1	11.94836888	Early nodulin 93 protein
Solyc06g050870.2	11.71262076	Hypoxia induced protein conserved region containing protein expressed
Solyc11g010990.1	11.49869951	Alcohol dehydrogenase
Solyc02g089140.2	10.99933479	B12D-like protein
Solyc12g089380.1	10.60827297	Expansin
Solyc02g083870.2	10.58637234	Gibberellin-regulated protein 2
Solyc05g046030.2	10.52629128	Peroxidase
Solyc11g040140.1	10.21034684	Xyloglucan endotransglucosylase/hydrolase
Solyc05g014710.2	10.18142015	Remorin family protein
Solyc04g005720.2	10.08058127	Calcium binding protein Caleosin
Solyc01g080790.2	10.07009862	PBSP domain-containing protein
Solyc06g059880.2	9.994506617	Acetolactate synthase
Solyc04g081860.2	9.937979658	Peroxidase
Solyc10g086520.1	9.854925669	Expansin-1
Solyc08g066590.2	9.805227296	Root cap protein 3
Solyc12g042930.1	9.727314366	Os01g0611000 protein
Solyc07g008420.2	9.68672101	Blue copper-like protein
Solyc11g069280.1	9.616419208	Root cap protein 1
Solyc01g079920.2	9.572532705	Xylanase inhibitor
Solyc05g055320.2	9.572532705	Peroxidase
Solyc05g014000.2	9.464620322	Pectate lyase
Solyc12g019140.1	9.415781131	Polygalacturonase
Solyc12g005020.1	9.365230493	Ring H2 finger protein
Solyc07g063800.1	9.365230493	Serine/threonine-protein kinase
Solyc01g090710.2	9.330518429	Malate dehydrogenase
Solyc02g067930.2	9.294950538	Glycerol-3-phosphate dehydrogenase
Solyc04g058180.2	9.294950538	Dehydration-responsive family protein
Solyc02g090540.2	9.258483552	Short-chain dehydrogenase/reductase family protein
Solyc03g097580.2	9.221070834	Bidirectional sugar transporter SWEET
Solyc01g009410.2	9.163067223	Peroxidase
Solyc09g014610.2	9.123060672	C4-dicarboxylate transporter/malic acid transport family protein

Solyc04g050170.2	9.060890538	Leucine-rich repeat receptor-like protein kinase PXL1
Solyc04g008470.2	9.039557205	Defensin protein
Solyc01g067370.2	8.950921995	TSB
Solyc02g082090.2	8.950921995	Peroxidase
Solyc03g121420.2	8.950921995	Phosphatase
Solyc01g068420.2	8.927885401	Os06g0207500 protein
Solyc08g005620.2	8.904474988	Gibberellin-regulated protein 7
Solyc10g078890.1	8.880678423	Peroxidase
Solyc05g051210.2	8.856482752	Os10g0578600 protein
Solyc04g049920.2	8.800749686	Probable RNA-binding protein ARP1
Solyc10g049660.1	8.755425761	Transposon protein Mutator sub-class
Solyc10g011730.2	8.729015369	Arabinogalactan peptide 20
Solyc04g064590.1	8.702112471	Protein kinase
Solyc05g025890.1	8.702112471	Acetyltransferase
Solyc05g005580.2	8.674698349	Expressed protein
Solyc05g043360.1	8.674698349	Laccase
Solyc02g093760.2	8.646753196	Tripartite motif-containing 25
Solyc06g035980.2	8.618256032	Actin depolymerizing factor 10
Solyc08g014560.1	8.618256032	Polygalacturonase
Solyc05g008550.2	8.559515306	Fruit-specific protein
Solyc02g080790.2	8.529223013	Deoxyhypusine synthase
Solyc01g100890.2	8.498281007	WAT1-related protein
Solyc02g080160.2	8.498281007	Xyloglucan endotransglucosylase/hydrolase
Solyc03g034110.2	8.498281007	Homeobox-leucine zipper protein ATHB-40
Solyc12g094470.1	8.466660804	Laccase-2
Solyc07g041640.2	8.434332007	Growth-regulating factor 1
Solyc07g044710.1	8.401262128	3-hydroxyisobutyryl-CoA hydrolase
Solyc08g080060.2	8.401262128	Expansin-like protein
Solyc12g007250.1	8.367416392	Xyloglucan endotransglucosylase/hydrolase
Solyc03g020070.2	8.367416392	Proteinase inhibitor II
Solyc07g055010.2	8.367416392	Metal ion binding protein
Solyc08g079080.2	8.345223111	Acid beta-fructofuranosidase
Solyc02g082940.2	8.332757519	Os03g0291800 protein
Solyc03g112790.2	8.297245472	Genomic DNA chromosome 5 TAC clone K6A12
Solyc06g074340.2	8.297245472	Hydrolase alpha/beta fold family protein
Solyc12g011030.1	8.290013363	Xyloglucan endotransglucosylase/hydrolase

Solyc10g008440.2	8.260837187	Expansin B1
Solyc08g080700.1	8.260837187	Carboxyl-terminal proteinase-like
Solyc10g080640.1	8.22348625	Calmodulin binding protein
Solyc07g064450.2	8.22348625	Cytochrome P450
Solyc08g008360.2	8.22348625	Unknown Protein
Solyc06g067910.2	8.192596695	Os01g0611000 protein
Solyc01g079370.2	8.145751869	GRAS transcription factor
Solyc07g043710.2	8.10525542	Acyltransferase
Solyc09g011820.2	8.10525542	Unknown Protein
Solyc05g052250.2	8.063589324	Expansin 2
Solyc03g059080.1	8.020684003	Receptor-like kinase
Solyc04g011470.1	8.020684003	CASP-like protein
Solyc06g084080.2	8.020684003	Guanylate-binding family protein
Solyc07g052440.2	7.976463477	Transmembrane protein 97
Solyc01g105350.1	7.930844556	Glycosyltransferase
Solyc02g079940.2	7.930844556	Unknown Protein
Solyc07g055180.2	7.883735903	Receptor protein kinase-like protein
Solyc09g098290.2	7.883735903	Receptor like kinase
Solyc12g010470.1	7.835036925	Zinc-binding protein
Solyc05g055200.2	7.835036925	Heat shock-like protein
Solyc06g068910.2	7.835036925	Receptor like kinase
Solyc10g078510.1	7.784636484	Glucan endo-1 3-beta-glucosidase 5
Solyc02g080910.2	7.784636484	Pectate lyase
Solyc03g033970.1	7.784636484	Phenylcoumaran benzylic ether reductase 3
Solyc07g045120.1	7.784636484	Exostosin family protein
Solyc08g042100.2	7.784636484	Armadillo/beta-catenin repeat family protein
Solyc09g014730.2	7.784636484	Receptor-like protein kinase
Solyc10g085380.1	7.732411371	Alcohol dehydrogenase
Solyc02g089350.2	7.732411371	Protein GAST1
Solyc06g066560.1	7.732411371	Aquaporin
Solyc01g099180.2	7.678224487	Lipoxygenase
Solyc01g106200.1	7.678224487	Oligopeptide transporter 4

Table S13. Top 100 DEGs up-regulated in "MgO7d vs. MgO7d+FOL21d" group.

Gene ID	log FC	Product
Solyc08g006850.2	14.35007894	Patatin
Solyc02g069190.2	11.76293902	Iron-regulated transporter 2
Solyc03g020070.2	11.44187194	Proteinase inhibitor II
Solyc09g082290.2	9.583832793	Non-specific lipid-transfer protein
Solyc02g085950.2	9.513537461	Ribulose bisphosphate carboxylase small chain 3B, chloroplastic
Solyc06g007970.2	9.469655159	Male sterility 5 family protein
Solyc04g006950.2	9.118231972	Unknown Protein
Solyc04g074170.1	8.777887168	MYB transcription factor
Solyc01g090890.2	8.486444071	Xenotropic and polytropic retrovirus receptor
Solyc11g012120.1	8.42649424	Plant-specific domain TIGR01615 family protein
Solyc08g007480.1	8.264721917	Cytochrome P450
Solyc07g055460.2	8.230070904	Cytochrome P450
Solyc03g044830.2	8.158167497	Guanine nucleotide-binding protein G(I)/G(S)/G(T) subunit beta-2
Solyc10g085360.1	8.002625363	Beta-fructofuranosidase insoluble isoenzyme 2
Solyc03g098720.2	7.960970627	Kunitz trypsin inhibitor
Solyc11g069240.1	7.918077351	Zinc finger family protein
Solyc08g066100.2	7.87386962	ATP-dependent 6-phosphofructokinase
Solyc02g086820.2	7.7928501	Carbonic anhydrase
Solyc05g051550.1	7.781170184	MYB transcription factor
Solyc04g010010.1	7.732486723	Thioredoxin-like protein 1
Solyc06g075150.2	7.732486723	Auxin response factor
Solyc01g065570.2	7.682102903	Chaperone protein dnaJ 49
Solyc09g092020.2	7.682102903	Probable calcium-binding protein CML22
Solyc03g113240.2	7.575727964	Unknown Protein
Solyc06g083930.1	7.519446895	LOB domain protein-like
Solyc11g006570.1	7.516764134	Inositol oxygenase
Solyc02g067850.2	7.4608808	Armadillo/beta-catenin repeat family protein
Solyc10g054070.1	7.39983625	Stylish 2b
Solyc12g010700.1	7.39983625	IST1 homolog
Solyc01g006080.1	7.336094171	Protein involved in mRNA turnover and stability (ISS)
Solyc10g047040.1	7.336094171	Zinc finger protein zfs1
Solyc11g013450.1	7.336094171	Unknown Protein

Solyc01g087980.2	7.269405091	Unknown Protein
Solyc10g084420.1	7.269405091	Unknown Protein
Solyc12g089300.1	7.269405091	Gibberellin-regulated protein 2
Solyc08g076700.1	7.199483245	MYB transcription factor
Solyc08g076980.2	7.199483245	Acetylornithine deacetylase or succinyl-diaminopimelate desuccinylase
Solyc11g071550.1	7.125999178	Glutamyl-tRNA(Gln) amidotransferase subunit A
Solyc03g006110.2	7.048570355	Non-specific serine/threonine protein kinase
Solyc03g006650.1	7.048570355	Solute carrier family 2, facilitated glucose transporter member 5
Solyc06g005850.2	7.048570355	Zinc finger family protein
Solyc02g078130.2	6.966749106	Basic helix-loop-helix family protein
Solyc05g005330.2	6.966749106	Cc-nbs-lrr, resistance protein with an R1 specific domain
Solyc06g074760.1	6.966749106	Zinc finger protein
Solyc10g079680.1	6.917537155	BHLH transcription factor-like
Solyc01g006440.1	6.88000695	Protein NRT1/ PTR FAMILY 7.1
Solyc11g071560.1	6.88000695	Restricted tev movement 2
Solyc02g070450.1	6.88000695	Glucan endo-1 3-beta-glucosidase 7
Solyc03g006100.2	6.88000695	Receptor like kinase
Solyc04g082280.2	6.88000695	AT1G76250-like protein
Solyc05g008950.2	6.88000695	Receptor-like kinase
Solyc06g048670.2	6.88000695	GDSL esterase/lipase
Solyc08g048290.2	6.88000695	Myo-inositol transporter 1
Solyc12g094730.1	6.787713887	AT-rich interactive domain-containing protein 3A
Solyc03g113180.2	6.787713887	class I heat shock protein 3
Solyc01g091400.2	6.68911061	LOB domain protein 31
Solyc01g102610.2	6.68911061	Ferric reductase oxidase
Solyc12g019180.1	6.68911061	Polygalacturonase 7
Solyc02g014070.1	6.68911061	ATP binding / serine-threonine kinase
Solyc09g074610.2	6.68911061	NAD(P)H-quinone oxidoreductase subunit T, chloroplastic
Solyc01g079460.2	6.583270501	Unknown Protein
Solyc01g111470.2	6.583270501	Pentatricopeptide repeat-containing protein
Solyc10g085210.1	6.583270501	BZIP family transcription factor
Solyc02g078720.2	6.583270501	Unknown Protein
Solyc06g084190.2	6.583270501	Non-specific lipid-transfer protein
Solyc09g084460.2	6.583270501	Chymotrypsin inhibitor 2

Solyc01g007870.2	6.469046548	Cyclin B-like protein
Solyc01g009480.2	6.469046548	Hydrolase alpha/beta fold family protein
Solyc01g094990.2	6.469046548	Glycosyltransferase-like protein
Solyc10g085440.1	6.469046548	Unknown Protein
Solyc03g044600.1	6.469046548	Unknown Protein
Solyc05g053520.2	6.469046548	DNA polymerase delta subunit 4 family
Solyc06g048620.2	6.469046548	Receptor like kinase
Solyc06g071400.2	6.469046548	Bidirectional sugar transporter SWEET
Solyc06g071650.1	6.469046548	AP-1 complex subunit mu
Solyc06g072310.2	6.469046548	Homeobox-leucine zipper protein PROTODERMAL FACTOR 2
Solyc06g083880.2	6.469046548	Unknown Protein
Solyc07g014750.1	6.469046548	Unknown Protein
Solyc08g008660.2	6.469046548	NAC domain-containing protein 12
Solyc01g099990.2	6.457414431	NAC domain protein
Solyc01g006520.2	6.344995394	Serine/threonine-protein kinase
Solyc01g009560.1	6.344995394	Terminal flower 1
Solyc11g071210.1	6.344995394	Unknown Protein
Solyc04g074840.2	6.344995394	Protein DETOXIFICATION
Solyc06g050360.2	6.344995394	Unknown Protein
Solyc07g053230.2	6.283723624	Myb-related transcription factor
Solyc11g011940.1	6.209265544	Homeobox-leucine zipper protein PROTODERMAL FACTOR 2
Solyc11g068970.1	6.209265544	Aluminum-activated malate transporter
Solyc02g071100.2	6.209265544	Purine permease family protein
Solyc02g085500.2	6.209265544	Ovate protein
Solyc03g033260.2	6.209265544	Maltose excess protein 1, chloroplastic
Solyc04g005700.2	6.209265544	Major latex-like protein
Solyc06g071190.2	6.209265544	IFA binding protein
Solyc09g084450.2	6.209265544	Chymotrypsin inhibitor-2
Solyc03g020080.2	6.119598466	Proteinase inhibitor II
Solyc01g060200.1	6.059427316	3&apos-5&apos exonuclease domain-containing protein-like
Solyc01g067230.2	6.059427316	Cullin 1-like protein C
Solyc12g007000.1	6.059427316	POT family domain containing protein expressed
Solyc02g021350.2	6.059427316	Testis-specific Y-encoded protein
Solyc02g036440.2	6.059427316	Cell cycle control protein

Table S14. Top 100 DEGs up-regulated in "H_2O7d+FOL1h vs. MgO7d+FOL1h" group.

Gene ID	log FC	Product
Solyc08g006850.2	9.090076	Patatin-like phospholipase family protein
Solyc10g075150.1	8.89116	Non-specific lipid-transfer protein
Solyc09g097810.2	8.780372	SAR8.2 protein
Solyc09g097760.2	8.494787	Glycine rich protein
Solyc12g096780.1	8.459263	Mitochondrial trans-2-enoyl-CoA reductase
Solyc03g098010.2	8.459263	Acid phosphatase
Solyc11g006570.1	8.182599	Inositol oxygenase
Solyc12g006280.1	7.783649	Myb-like DNA-binding domain SHAQKYF class family protein
Solyc12g056750.1	7.725028	WRKY transcription factor 7
Solyc01g090890.2	7.663924	Xenotropic and polytropic retrovirus receptor
Solyc06g008560.1	7.663924	Os03g0859900 protein (Fragment)
Solyc10g039390.1	7.230333	ATFP3 (Fragment)
Solyc10g079860.1	7.230333	Beta-1 3-glucanase
Solyc10g005390.2	7.143471	Linalool synthase
Solyc07g007250.2	7.051042	Metallocarboxypeptidase inhibitor
Solyc01g101190.2	6.846263	Alpha-humulene/(-)-(E)-beta-caryophyllene synthase
Solyc03g025670.2	6.787005	PAR-1c protein
Solyc09g097770.2	6.742664	Cell wall protein
Solyc10g079680.1	6.731831	BHLH transcription factor-like
Solyc12g088280.1	6.731831	Serine carboxypeptidase family protein
Solyc08g013670.2	6.731831	Photosystem I reaction center subunit
Solyc09g082300.2	6.731831	Non-specific lipid-transfer protein
Solyc09g082460.2	6.731831	Homocysteine s-methyltransferase
Solyc01g101170.2	6.607533	Viridiflorene synthase
Solyc12g096900.1	6.607533	Tir, resistance protein fragment
Solyc01g005900.2	6.471508	Transferase family protein
Solyc01g099990.2	6.471508	F-box protein PP2-B1
Solyc03g044830.2	6.471508	Guanine nucleotide-binding protein G(I)/G(S)/G(T) subunit beta-2
Solyc08g007770.2	6.471508	PGR5-like protein 1A, chloroplastic
Solyc09g084460.2	6.471508	Chymotrypsin inhibitor 2

Solyc01g101180.2	6.321311	Alpha-humulene/(-)-(E)-beta-caryophyllene synthase
Solyc03g005650.1	6.321311	Cc-nbs-lrr, resistance protein
Solyc02g077040.2	6.202824	Cathepsin B-like cysteine proteinase 5
Solyc12g009130.1	6.153639	Unknown Protein
Solyc03g006210.1	6.153639	Cathepsin B-like cysteine proteinase
Solyc06g072320.2	6.153639	8-amino-7-oxononanoate synthase
Solyc07g052370.2	6.153639	Cytochrome P450
Solyc07g063600.2	6.153639	Chlorophyll a-b binding protein, chloroplastic
Solyc09g011270.2	6.153639	CHP-rich zinc finger protein-like
Solyc10g047660.1	5.963886	CHCH domain containing protein expressed
Solyc12g005680.1	5.963886	Potassium transporter
Solyc12g077460.1	5.963886	Unknown Protein
Solyc02g069460.2	5.963886	Photosystem I reaction center subunit III
Solyc05g008960.2	5.963886	Receptor-like protein kinase
Solyc05g009620.2	5.963886	Cell number regulator 3
Solyc05g051270.2	5.963886	Cysteine-rich repeat secretory protein 60
Solyc05g052240.2	5.963886	Chalcone--flavonone isomerase
Solyc08g021940.1	5.963886	Plant synaptotagmin
Solyc08g065710.1	5.963886	Vacuolar processing enzyme
Solyc01g005040.2	5.745341	CASP-like protein
Solyc01g095560.2	5.745341	Unknown Protein
Solyc10g075070.1	5.745341	Non-specific lipid-transfer protein
Solyc10g079660.1	5.745341	Importin alpha-1b subunit
Solyc10g085240.1	5.745341	Glycosyltransferase
Solyc06g034000.1	5.745341	Myb-related transcription factor-like protein
Solyc06g066640.2	5.745341	Photosystem I reaction center subunit VI-1, chloroplastic
Solyc07g018310.2	5.745341	Unknown Protein
Solyc08g014230.2	5.745341	2-isopropylmalate synthase
Solyc09g014860.2	5.745341	Os03g0816700 protein
Solyc01g088620.2	5.487675	Ferredoxin-like
Solyc10g054040.1	5.487675	26S protease regulatory subunit
Solyc11g006220.1	5.487675	Myosin XI
Solyc12g094660.1	5.487675	Cc-nbs-lrr, resistance protein
Solyc02g078290.2	5.487675	Myosin-like protein

Solyc02g082640.1	5.487675	Unknown Protein
Solyc04g008000.2	5.487675	Calmodulin
Solyc05g006440.2	5.487675	Homology to unknown gene
Solyc05g008110.2	5.487675	Unknown Protein
Solyc05g053150.1	5.487675	MYB transcription factor
Solyc06g054520.1	5.487675	3-hydroxyisobutyryl-CoA hydrolase
Solyc06g068900.2	5.487675	Nudix hydrolase 4
Solyc07g049670.2	5.487675	Alcohol acetyltransferase
Solyc07g052880.2	5.487675	Formin 3
Solyc07g056180.1	5.487675	NBS-LRR class disease resistance protein
Solyc08g076970.2	5.487675	Acetylornithine deacetylase or succinyl-diaminopimelate desuccinylase
Solyc08g080460.1	5.487675	Serine/threonine protein kinase
Solyc09g082810.2	5.487675	Unknown Protein
Solyc01g097240.2	5.29639	Pathogenesis-related protein 4B (Fragment)
Solyc01g006200.2	5.173763	Unknown Protein
Solyc01g007830.1	5.173763	Unknown Protein
Solyc01g009560.1	5.173763	Terminal flower 1 (Fragment)
Solyc01g009580.1	5.173763	Terminal flower 1 (Fragment)
Solyc01g014270.2	5.173763	Unknown Protein
Solyc10g009520.2	5.173763	Dihydroflavonol-4-reductase
Solyc10g054070.1	5.173763	Stylish 2b
Solyc10g075110.1	5.173763	Non-specific lipid-transfer protein
Solyc10g081890.1	5.173763	Aluminum-activated malate transporter (Fragment)
Solyc11g005440.1	5.173763	Limkain b1 (Lkap) (Fragment)
Solyc11g018590.1	5.173763	Subtilase family protein
Solyc11g042950.1	5.173763	Carbohydrate kinase FGGY
Solyc12g098930.1	5.173763	Pyruvate dehydrogenase kinase
Solyc02g062490.2	5.173763	2-oxoglutarate-dependent dioxygenase (Fragment)
Solyc02g063180.2	5.173763	UDP-N-acetylenolpyruvoylglucosamine reductase
Solyc02g067350.2	5.173763	cDNA clone J023065D24 full insert sequence
Solyc02g080520.2	5.173763	Os04g0625000 protein
Solyc02g084590.2	5.173763	GATA transcription factor 9
Solyc02g086990.2	5.173763	Cyclic nucleotide gated channel
Solyc08g006850.2	9.090076	Patatin-like phospholipase family protein
Solyc10g075150.1	8.89116	Non-specific lipid-transfer protein

Solyc09g097810.2	8.780372	SAR8.2 protein
